# Unlocking the Potential Use of Reactive POSS as a Coagent for EPDM/PP-Based TPV

**DOI:** 10.3390/polym15102267

**Published:** 2023-05-11

**Authors:** Nazlı Yazıcı Çakır, Özgenur İnan, Merve Ergün, Mehmet Kodal, Güralp Özkoç

**Affiliations:** 1 Department of Chemical Engineering, Kocaeli University, 41001 Kocaeli, Turkey; nazliyazici93@gmail.com (N.Y.Ç.); mervemetin1576@gmail.com (M.E.); 2 Polymer Science and Technology Graduate Program, Kocaeli University, 41001 Kocaeli, Turkey; inannozgee@gmail.com; 3 Nanotechnology Research and Application Center, Sabancı University, 34956 Istanbul, Turkey; 4 Department of Chemistry, Istinye University, 34396 Istanbul, Turkey; 5 Xplore Instruments B.V., 6135 KT Sittard, The Netherlands

**Keywords:** thermoplastic vulcanizates, dynamic vulcanization, coagents, POSS

## Abstract

Thermoplastic vulcanizates (TPVs) are multifunctional materials consisting of two or more phases with solid elastomeric properties at room temperatures and fluid-like properties above their melting point. They are produced through a reactive blending process known as dynamic vulcanization. The most widely produced TPV type is ethylene propylene diene monomer/polypropylene (EPDM/PP), which is the focus of this study. The peroxides are mainly selected to be used in crosslinking of EPDM/PP-based TPV. However, they still have some disadvantages, such as the side reactions resulting in the beta chain scission of the PP phase and undesired disproportionation reactions. To eliminate these disadvantages, coagents are used. In this study, for the first time, the use of vinyl functionalized polyhedral oligomeric silsesquioxane (OV-POSS) nanoparticles was investigated as a potential coagent in EPDM/PP-based TPV production via peroxide-initiated dynamic vulcanization. The properties of the TPVs having POSS were compared with the conventional TPVs containing conventional coagents, such as triallyl cyanurate (TAC). POSS content and EPDM/PP ratio were investigated as the material parameters. Mechanical properties of EPDM/PP TPVs exhibited higher values in the presence of OV-POSS, which resulted from the active participation of OV-POSS into the three-dimensional network structure of EPDM/PP during dynamic vulcanization.

## 1. Introduction

Thermoplastic vulcanizates (TPVs) are multifunctional polymeric materials classified as thermoplastic elastomers. TPV materials comprise a large amount of crosslinked elastomer as a dispersed phase and a small amount of continuous thermoplastic matrix [1,2]. Therefore, they combine the high elasticity of conventional rubbers and the excellent processability and recyclability of thermoplastics. Consequently, TPVs have attracted considerable attention and are widely used in industrial applications, such as automotive, white goods, electronics, and other sectors being ‘greener’ than cross-linked conventional elastomer materials [3]. Although many different types of TPV are produced, such as polypropylene/ethylene octene copolymer (EOC), PP/natural rubber (NR), polyethylene (PE)/NR, PP/butyl rubber (IIR), etc., the most common and commercially used is the EPDM/PP TPV system. Dynamic vulcanization (DV) is a complex polymer reactive blending technique in TPV production [4,5,6,7,8,9,10,11,12,13,14,15,16,17]. In this technique, crosslinking and breakup of the rubber phase occur simultaneously, resulting in phase inversion during dynamic vulcanization [18].

The most widely used crosslinking agents for EPDM/PP TPVs are peroxides and phenolic resins [19,20,21]. Dynamically vulcanization of the EPDM phase in the EPDM/PP system with peroxides has been well-known for many years. Low compression set values and high-temperature resistance of final products are the general advantages of crosslinking TPVs with peroxides. In addition, the vulcanization of rubbers through unsaturated and saturated bonds can be carried out with a peroxide [4,22]. However, it has some disadvantages, such as sensitivity to oxidation during crosslinking and unintended reactivity towards other constituents, such as oils, antioxidants, and some fillers. Moreover, PP undergoes β-chain scission reactions during dynamic vulcanization, which leads to a decrease in the molecular weight of PP.

Coagents are used to eliminate these disadvantages in the presence of peroxides [4,22,23,24,25]. Coagents make crosslink bridges between elastomer chains and increase the crosslinking efficiency of peroxide by decreasing the unwanted beta chain scissions and disproportionate termination reactions [26,27]. The properties of the crosslinked structure to be obtained are also directly related to the type of coagent used [28]. The influence of different coagents on the properties of EPDM/PP-based TPVs was extensively studied in the literature. Researchers examined the influence and effectiveness of coagents having different structures, such as triallyl cyanurate (TAC), triallyl isocyanurate (TAIC), trimethylolpropane triacrylate (TMPTA), N,N′-m-phenylene dimaleimide (MPDM). Among the coagents investigated, the compounds containing TAC exhibited better mechanical properties and also showed the highest elastic recovery compared to other coagent systems [9,29,30,31,32,33]. Therefore, the TAC coagent was used to produce TPVs as the control and named as the reference in this study.

Polyhedral oligomeric silsesquioxanes (POSSs) are structurally lattice-shaped molecules. It can be called polyhedral skeletons formed by silicon and oxygen with closed formulas (RSiO1.5). Here, “n” is greater than four and is often 8. The R group in the structure can consist of many different functional groups (vinyl, hydroxyl, epoxy, amine, etc.). Since POSSs are 1.5 nm in size and have an average molecular weight of 1000 Da, they are almost similar in size to many polymer molecules. Recent studies show that different types of POSS molecules have been used as co-plasticizer, nucleating agents, adhesion promoters, non-flammability additives, compatibilizers, modification agents, antibacterial agents, anti-proliferative agents, etc. [34,35,36,37,38,39,40,41,42,43,44,45,46].

POSS structures containing vinyl or acrylic groups can turn into very reactive coagents in the presence of peroxides. Therefore, it can increase crosslinking efficiency of peroxide-containing TPV systems. Blanco and Zaharescu prepared EPDM/POSS composites and subjected them to γ-irradiation. They stated that the POSS cage restricted the chain mobility of EPDM [47]. In a recently published study, a novel structure of norbornene reactive Double-Decker silsesquioxanes (DN-DDSQ) was designed and synthesized to covalently bonded into the EPDM molecular chain segment by the crosslinking reaction to form a denser cross-linked network and introduce a high-temperature resistant cage structures in EPDM to improve the crosslinking, mechanical, heat-shielding, and ablation properties of EPDM. The authors reported that the resultant formation of a denser cross-linked network and better dispersion of the high-temperature resistant cage structure of DDSQ in the EPDM ablation resistance composites improved its mechanical and ablation performance [48]. Morici et al. reported that a POSS type with vinyl groups was used to crosslink polyethylene with peroxides [49]. In this study, POSSs and PE were mixed with dicumyl peroxide (DCP) with one-step reactive melt mixing. The results show that the presence of DCP in the system affects the successful crosslinking reactions. In another study, for the crosslinking of low-density polyethylene (LDPE), DCP-activated octavinyl-POSS was used as a crosslinker [50]. The crosslinking rate increased with DCP content, while the degree of crosslinking increased with POSS content. Therefore, it was emphasized that an optimal combination of POSS-DCP is required for better crosslinking. Crosslinking led to an increase in storage modulus and a decrease in the thickness of the lamellar crystal of polyethylene. In a previous study, POSS molecules with vinyl and acrylic functional groups were used as coagents to cross-link natural rubber (NR) with sulfur [41,42]. The results showed that the crosslink density and crosslink reaction rate increase in the presence of POSS. In addition, the mechanical and physical properties obtained in the compounds using POSSs are relatively developed. In another study, different types of polyethylene (LDPE, HDPE, and LLDPE) were cross-linked by using POSS containing -C=C- bonds in the presence of peroxide [51,52]. The studies show that POSSs are more reactive and efficient than conventional coagents. The mechanical properties of crosslinked polyethylenes prepared using POSSs were better obtained than those prepared using other coagents. POSSs containing -C=C- double bonds in their structure that can participate in crosslinking have the potential to be used as a coagent in TPV production.

In this study, an octavinyl functionalized POSS (OV-POSS) was utilized as a coagent for the first time in a TPV system. The vinyl groups on the nanocage of OV-POSS would make it involved in the crosslinking reaction as a coagent and simultaneously improve the strength of the resulting TPV at a molecular level due to the molecular stiffness of the POSS cage. Dynamic vulcanization in the presence of peroxide was carried out in a laboratory micro-compounder (MC15 Xplore Instruments, Sittard, The Netherlands). The content of OV-POSS as a coagent, peroxide content, and EPDM/PP ratio was taken as the material parameters. The crosslinking density, compression set, tensile properties, morphology, and rheology of the TPVs were investigated. The performance of nascent TPV was compared with that of conventional TPVs.

## 2. Materials and Methods

### 2.1. Materials

The materials used in the study are shown in Table 1. The literature indicates that PP homopolymers with melt flow index (MFI) values between 1–12 are generally chosen for EPDM/PP TPVs [1,18,53,54]. The critical point here is that the viscosity values of EPDM and PP must be close to each other at process conditions to ensure a good blending. Although the viscosity value of EPDM changes according to the oil content, its viscosity generally is higher than that of commodity thermoplastics. For this reason, it is more appropriate to use a PP having a relatively low MFI value, in other words, a high viscosity. The EPDM used in the study has 50% paraffin oil content, a typical type of EPDM used industrially in TPV production. OV-POSS and antioxidants (Irganos^®^ and Irgafos^®^) were purchased from Hybrid Plastics and BASF, respectively.

### 2.2. Processing

**Figure 1:**
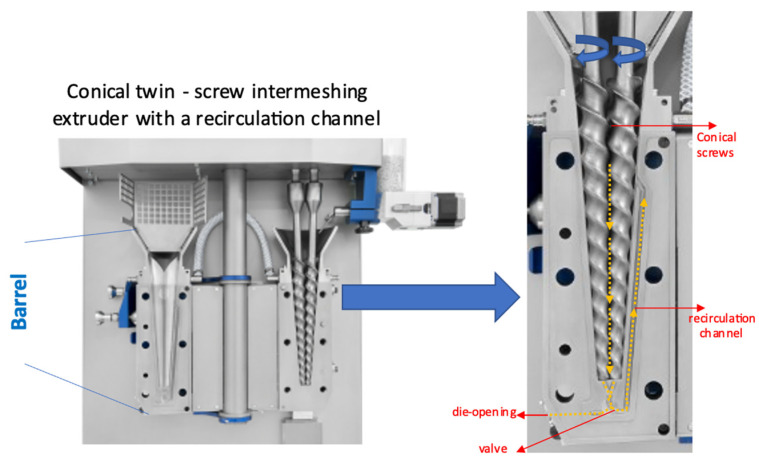
MC 15 HT twin screw microcompounder (Xplore Instruments, Sittard, The Netherlands).

TPVs were prepared via melt blending in a vertically positioned twin-screw batch micro-compounder having a recirculation channel (15 mL micro-compounder—MC 15 HT, Xplore Instruments, Sittard, The Netherlands) (Figure 1). The barrel temperature of the microcompounder was set to 175 °C. The compounding time was kept constant at 5 min. DCP was added to the system at the 3rd minute. At the end of the mixing period, the extrudate was transferred to the injection molding device (12 mL Xplore Injection molding machine). The molten compound was subsequently injection molded to obtain standard test samples. The mold and melt temperatures were 25 °C and 175 °C, respectively. The injection and holding pressures were set to 8 bars.

The coagent/DCP ratio was taken as 1, 3, 5, and 7. The samples were abbreviated with respect to the type and loading level of the coagent, such as 70/30 EPDM/PP/Coagent(X) (Table 2). Here, X stands for the coagent/DCP ratio. For instance, when X is equal to 5, coagent content then becomes 5 times higher than that of DCP. Moreover, DCP content was kept constant at 1.75, 1.50, 1.25, 1.00, and 0.75 phr for a given EPDM/PP ratio, as seen in Table 2. For instance, DCP content is 1.75 phr for 70/30 EPDM/PP sample, including 70 phr EPDM.

### 2.3. Characterization

An Anton Paar Modular Compact Rheometer, MCR 102, with plate-plate geometry of 25 mm diameter, was used to conduct the rheological tests. Frequency sweep measurements were carried out at 175 °C under a nitrogen atmosphere at a shear strain of 1% and an angular frequency range between 0.1 and 600 rad/s.

The tensile properties were measured with an Instron Universal Testing Machine (Model 3345) according to ISO37 with type 2 sample geometry. The crosshead speed was 500 mm/min. Tensile strength, elongation at break, and 100% modulus values of TPVs were obtained from stress–strain graphs.

Compression set determination of the samples was conducted according to ASTM D395 with method B and type 2 geometry. The hardness of the samples was measured according to ASTM D2240 using a Zwick Shore-type durometer.

The overall crosslink density (CLD) of the EPDM phase in EPDM/PP TPVs was determined based on solvent-swelling measurements in cyclohexane at 25 °C according to the Flory–Rehner method (Equation (1)). Samples were submerged in acetone, then cyclohexane. After 24 h, the swollen sample was weighed, dried, and weighed again. From the degree of swelling, an overall CLD was calculated relative to the EPDM + PP phases as expressed by *Ʋ*_e_ + PP. The latter was done to avoid the need to correct for a part of the PP being extracted as amorphous PP [55,56,57].

(1) $$({Ʋ}_{e}+\mathrm{PP})=\frac{\rho }{M_{c}}=\frac{-[\ln\left(1-\;\varphi \right)+\varphi ꭕ\varphi^{2}]}{{{(V}_{o}\varphi )}^{1/3}}$$

where “M_c_” is the average molecular weight between the crosslinked points (g/mol), “ρ” is the density of samples (g/cm^3^), “V_0_” is the molar volume of the solvent (for cyclohexane is 108.7 cm^3^/mol), “ꭕ” is polymer-swelling agent interaction parameter, or Flory–Huggins parameter, which in this case is 0.315 [28], and “φ” volume fraction of EPDM in the swollen network, which is expressed by Equation (2):

(2) $$\varphi =\frac{\frac{W_{0}}{\rho }}{\frac{W_{s}-{\;W}_{0}}{\rho_{1}}+\frac{W_{0}}{\rho }}$$

where “W_0_” is the initial mass of the sample (g), “W_s_” is the mass of the swollen sample (g), and “ρ_1_” and “ρ” are the density of the cyclohexane and EPDM (g/cm^3^), respectively. Sample densities were measured with the Mettler Toledo density kit according to the Archimedes principle.

Swelling measurements were carried out to estimate the oil resistance according to ASTM D741. The mass change was measured after swelling in IRM903 standard oil for 70 h at 125 °C and dried for an hour at ambient temperature. The change in mass (Δm) can be calculated from Equation (3) using the final (mf) and initial (mi) weights of the specimen.

(3) $$\Delta m\;\left(\%\right)=\left[\left(\mathrm{mf}-\mathrm{mi}\right)/\mathrm{mi}\right]\times 100$$

The surface morphologies of the TPVs were examined by the QUANTA 400F Field Emission model scanning electron microscope (SEM) to determine the phase sizes and interfacial interactions of the samples. The samples were cryogenically broken for SEM analysis, and the fracture surfaces were sputter-coated with gold. Moreover, the surface morphology of TPVs was also evaluated by atomic force microscopy (AFM, Nanosurf model) in dynamic force mode. A microtome was used to prepare ultrathin sections for AFM analyses.

## 3. Results and Discussions

### 3.1. Rheological Properties of the TPVs

**Figure 2:**
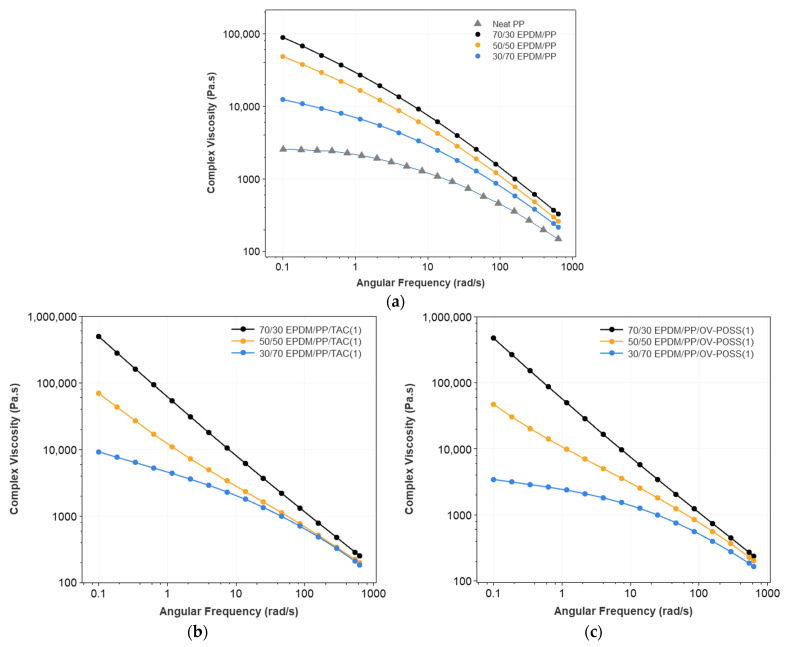

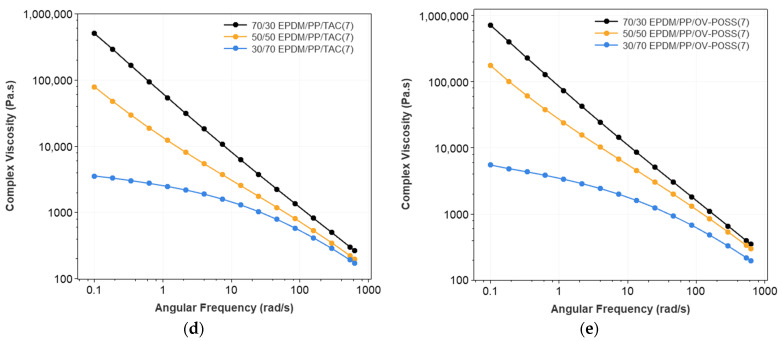
Complex viscosity of (**a**) neat PP, EPDM/PP blends with respect to the different EPDM/PP composition and EPDM/PP TPVs as a function of EPDM/PP ratio and coagent types (**b**) EPDM/PP/TAC(1), (**c**) EPDM/PP/OV-POSS(1), (**d**) EPDM/PP/TAC(7), and (**e**) EPDM/PP/OV-POSS(7).

**Figure 3:**
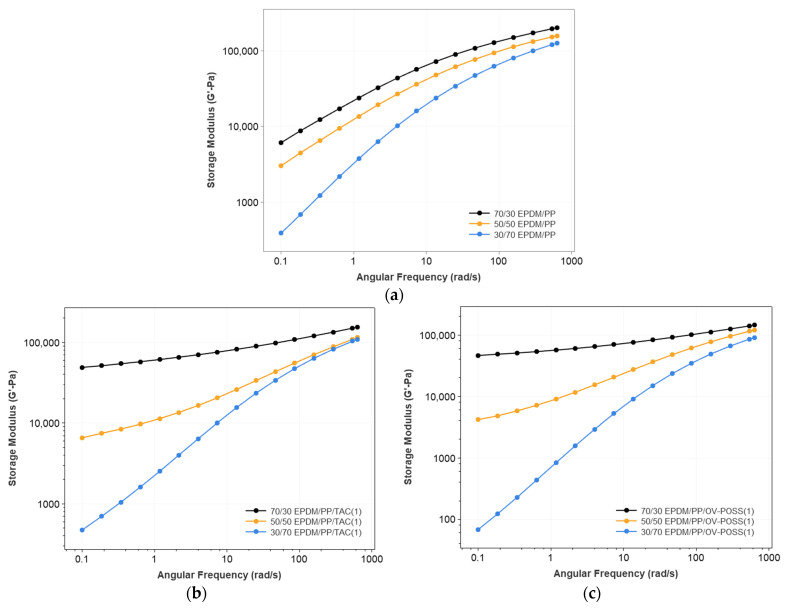

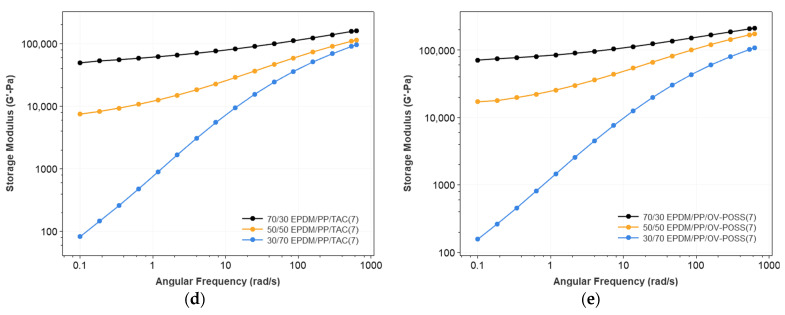
The storage modulus of (**a**) neat PP, EPDM/PP blends with respect to the different EPDM/PP composition and EPDM/PP TPVs as a function of EPDM/PP ratio and coagent types (**b**) EPDM/PP/TAC(1), (**c**) EPDM/PP/OV-POSS(1), (**d**) EPDM/PP/TAC(7), and (**e**) EPDM/PP/OV-POSS(7).

**Figure 4:**
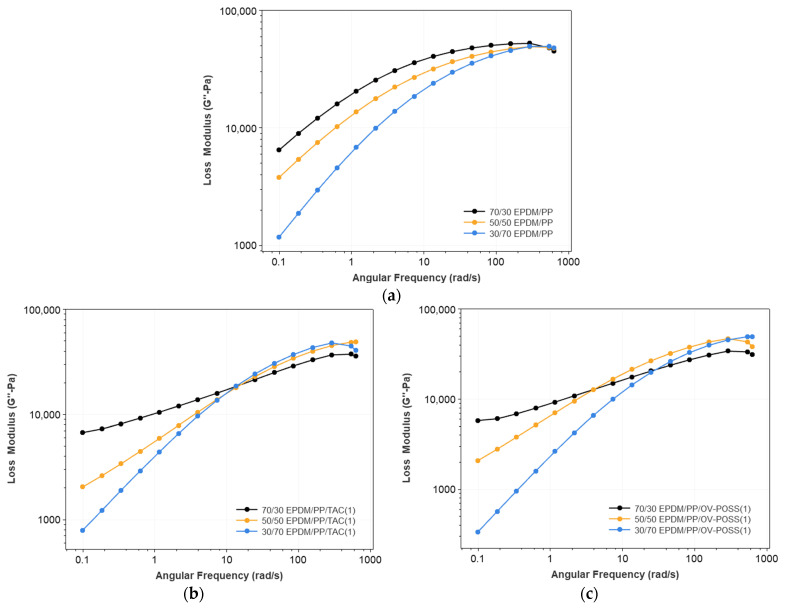

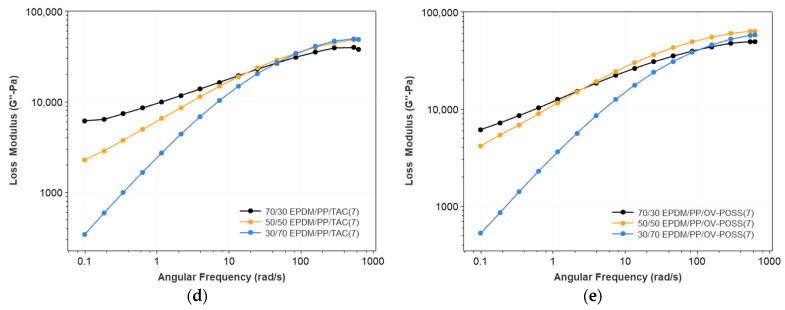
The loss modulus of (**a**) neat PP, EPDM/PP blends with respect to the different EPDM/PP composition and EPDM/PP TPVs as a function of EPDM/PP ratio and coagent types (**b**) EPDM/PP/TAC(1), (**c**) EPDM/PP/OV-POSS(1), (**d**) EPDM/PP/TAC(7), and (**e**) EPDM/PP/OV-POSS(7).

The variation of complex viscosity (η*), storage (G′), and loss (G″) modulus concerning the EPDM/PP ratio and content of the TAC and OV-POSS as the coagent are shown in Figure 2, Figure 3, Figure 4 and Figure S1–S3.

All the complex viscosity values (η*) decreased with increasing frequency (ω), indicating the shear-thinning behavior of the pseudoplastic liquid due to the deformation of the entanglement of the chains at high frequencies. The neat-PP exhibited a plateau region at low shear rates, where complex viscosity is slightly influenced by the increasing frequency, followed by a decreasing viscosity region at high shear rates due to the alignment of the molecules (Figure 2a). Similar behavior was also observed in 30/70 EPDM/PP blends independently from the content of OV-POSS. This can be attributed to the phase morphology of the TPV, where PP is the continuous phase and dominates the viscoelastic behavior. The apparent difference between coagent (TAC and OV-POSS) containing and non-containing TPVs is the linear dependency of the log(η*) to the log(ω) for 50/50 and 70/30 EPDM/PP TPV systems. This indicates that EPDM/PP melt-flow under controlled shear conditions obeys the power law above 1 rad/s. It can be seen that samples having high EPDM and coagent concentrations showed a pronounced viscosity up-turn at low shear rates (w < 1 rad/s). The rheological behavior of this dynamically vulcanized TPV is similar to those of block copolymers and highly particle-filled composites [58]. This can be attributed to the 3D network structures formed in the cured rubber particle and the hyperbranched PP matrix due to possible radical grafting reactions onto OV-POSS and rubber-PP grafting reactions. Increasing coagent content resulted in higher complex viscosities due to the enhanced crosslinking efficiency of the rubber phase, which enhances the elastic behavior of the resulting TPV. Compared to TPVs containing OV-POSS, TPVs containing TAC appears to have higher viscosities at low frequencies due to the higher crosslink density.

It is known that the TPV’s morphology and structure influence the rheological behavior. The viscoelastic properties of a binary polymer blend system depend on the dispersed phase’s deformability and shape/size. Moreover, the deformability is also related to the size of the dispersed component. The elastic and viscous characteristics of the TPVs are well reflected by the change of G′ and G″ with angular frequency. Generally, at low frequencies, the G′ value provides information about the long-range relaxation (beyond entanglement distance). In contrast, the corresponding value at high frequency relies on short-range relaxation (motion with entanglement) [59]. Figure 3 and Figure 4 show the variation of G′ and G″ values for selected compositions with frequency. It is seen that the values of G′ and G″ increase monotonously with increasing angular frequency for all blends. In addition, the increasing EPDM content resulted in higher G′ and G″ values in nearly the entire range of angular frequency values. However, depending on the EPDM/PP ratio and the coagent content, the rate of increase (the slope of the G′(angular frequency)) changed. One should note that at low frequencies, the elasticity of the TPV was controlled by the rubber phase, but at higher frequencies, the elasticity was dominated by the PP phase [60,61]. Without a coagent, the slope of log(G′) vs. log(ω) was constant below 10 rad/s indicating a linear increase in G′ concerning ω; however, above 10 rad/s, the slope became smaller and approached zero. The G″ vs. angular frequency curves also approached zero. This shows that in the absence of a coagent, the network formed due to dynamic vulcanization can still undergo a relaxation at high frequencies. In addition, in the lack of a coagent, the G′’ values were more petite or nearly the same as G′ values, pointing out that the TPVs had a viscosity. When only 1% OV-POSS was added to the TPV system, the character of the G′ vs. G″ curves changed due to the efficient dynamic crosslinking. In this case, especially for 50% and 70% EPDM-containing systems, the slope of the log(G′) vs. log(angular frequency) is minimal (even approaches zero) at lower frequencies (angular frequency < 1 rad/s). As the shear rate increased, the G′ values started to rise with a higher slope. The reason is that both the network formation in the rubber domains and the rubber-PP phase hinder the relaxation process in the presence of a coagent. This is more pronounced in the presence of 7% coagent, especially OV-POSS. In this case, due to the highly entangled network of rubber domains and rubber-PP in the presence of OV-POSS compared to TAC, the relaxation is suppressed, and the elasticity of the TPV becomes dominant.

### 3.2. Mechanical Properties and Crosslink Density of the TPVs

In a TPV system, the mechanical properties are associated with the rubber/plastic ratio, the crosslink density of the rubber phase, the size and distribution of the rubber phase, the thickness of the plastic ligaments, and the compatibility of the plastic and rubber phases [62,63,64,65,66]. When the studies in the literature were examined, it was observed that the tensile strength and hardness values increased with the increasing content in the thermoplastic phase ratio [62]. The crosslink density is one of the essential properties of elastomeric materials, and the mechanical properties of elastomers largely depend on the crosslink density of the elastomer phase [67,68]. The tensile strength and elongation at break values increase when crosslinking of EPDM reaches an optimum value, and the crosslinked rubber particles are homogeneously dispersed in the continuous phase in the EPDM/PP TPV systems. However, at very high crosslink densities, the mechanical properties are adversely affected due to the coarse distribution of the crosslinked rubber particles that could act as stress transfer in the matrix [69]. Therefore, the optimum crosslink density value should be sustained to obtain higher mechanical properties [62,70]. In addition, the particle size of the dispersed phase is important in this context [62,69].

The mechanical properties of nano-sized particle-reinforced polymer composites, such as POSS nanoparticle-containing polymer composites, depend highly on particle size, particle-matrix interfacial adhesion, and particle loading ratio [71]. Rigid inorganic particles often have higher stiffness than the polymer matrix. Therefore, the elastic modulus of the matrix can be improved with these nano or micro-sized particles [72,73,74,75,76]. However, the tensile strength of the material depends on the stress transfer that takes place between the particle and the matrix. In the case of well-bonded particles, the stress applied to the matrix is effectively transferred from the matrix to the particles [77]. This causes a noticeable increase in strength values [78,79,80,81,82]. However, micro or nanoparticles poorly bonded to the matrix cause a decrease in the strength of the material [83,84,85,86]. On the other hand, the most critical factor for the mechanical properties of TPVs is the homogeneous distribution of the crosslinked three-dimensional network.

**Figure 5:**
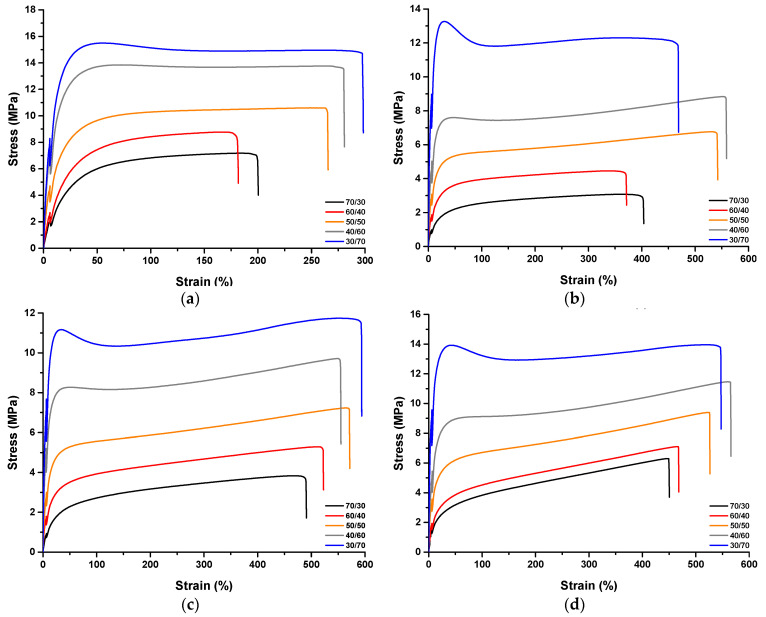
Representative stress–strain curves of EPDM/PP and EPDM/PP TPVs as a function of EPDM/PP ratio and coagent types (**a**) EPDM/PP, (**b**) EPDM/PP + DCP, (**c**) EPDM/PP/TAC(3), and (**d**) EPDM/PP/OV-POSS(3).

The representative stress–strain curves of selected TPVs are given in Figure 5 and Figure S4. As can be seen, samples of EPDM/PP TPVs with high PP ratios exhibited higher mechanical properties, such as modulus and yield strength. The yield point and necking behavior were followed by a cold drawing before the fracturing in TPVs with high PP content. However, the neck formation was not observed with increasing EPDM, and the samples showed lower mechanical properties. A possible reason for reducing mechanical properties, such as elongation at break and 100% modulus, could be the chain scission reactions that would cause the molecular weight loss in PP with the addition of peroxide (EPDM/PP + DCP) independent of the mixing ratio to the EPDM/PP TPV. With the use of TAC and especially OV-POSS as a coagent, increases in the mechanical properties of EPDM/PP TPVs were observed with the rise in crosslink efficiency. EPDM/PP/OV-POSS TPVs exhibited relatively higher mechanical properties. This can be attributed to the formation of several crosslink bridges through the unsaturated double bonds of octavinyl groups on the highly stiff POSS cage that formed the links of a self-reinforced network structure. These findings are also consistent with the crosslink density test results given in Table 3 in the upcoming part.

**Figure 6:**
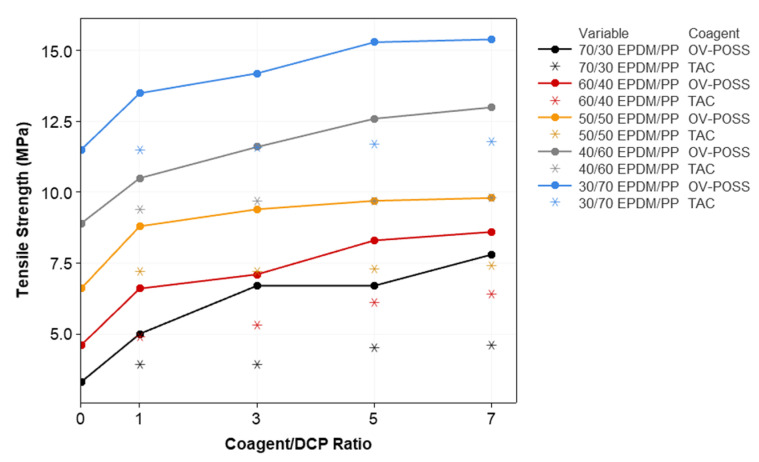
The variation in the tensile strength values of EPDM/PP TPVs with respect to EPDM/PP ratios, coagent types, and crosslinker system ratios.

**Figure 7:**
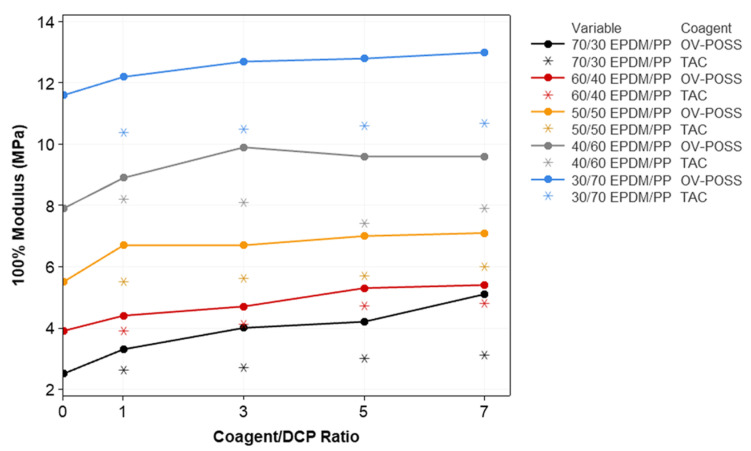
The variation in the 100% modulus values of EPDM/PP TPVs with respect to EPDM/PP ratios, coagent types, and crosslinker system ratios.

**Figure 8:**
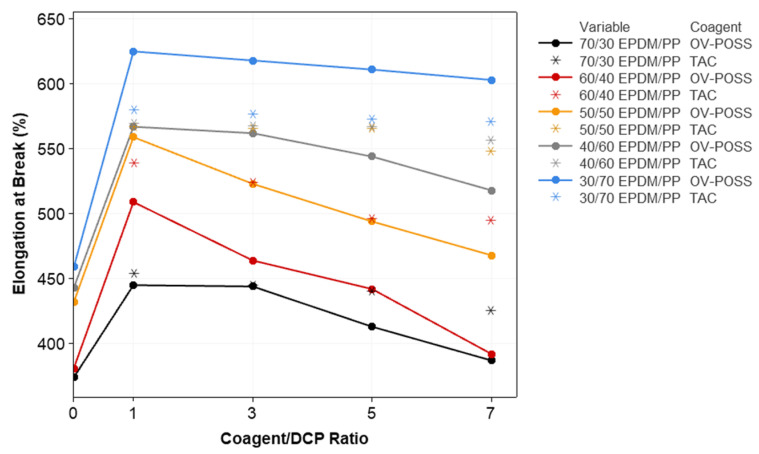
The variation in the elongation at break values of EPDM/PP TPVs with respect to EPDM/PP ratios, coagent types, and crosslinker system ratios.

**Figure 9:**
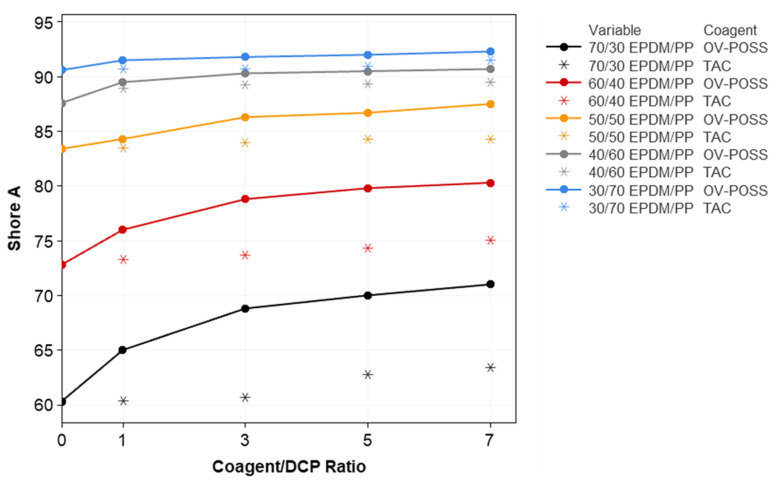
The variation in the Shore A values of EPDM/PP TPVs with respect to EPDM/PP ratios, coagent types, and crosslinker system ratios.

**Figure 10:**
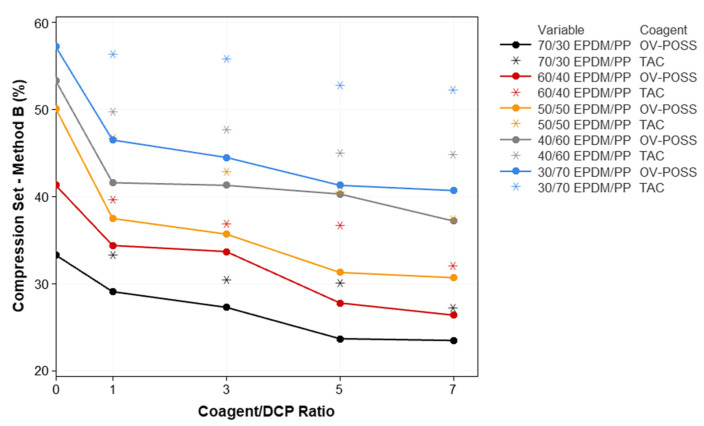
The variation in the compression set values of EPDM/PP TPVs with respect to EPDM/PP ratios, coagent types, and crosslinker system ratios.

The variation in the mechanical properties of the samples, such as elongation at break, 100% modulus, tensile strength, compression set, and hardness with respect to the composition, is given in Figure 6, Figure 7, Figure 8, Figure 9 and Figure 10. It is seen that, especially when OV-POSS is used as a coagent, the tensile strength and the 100% modulus values are higher than that of the TPVs prepared with TAC (Figure 6 and Figure 7). This can be attributed to the participation of OV-POSS in the crosslinking reactions, which resulted in improved mechanical properties. In addition, in the presence of OV-POSS having eight unsaturated vinyl groups per molecule, the chain scission reactions of PP were suppressed, resulting in higher mechanical properties. In addition, the possibly formed EPDM-OVPOSS-PP graft structures could act as a compatibilizer in EPDM/PP interphase and lead to phase compatibility. They could also bring some level of physical crosslinking in the structure of TPV. Therefore, the tensile strength and 100% modulus values increased. Similar findings were also reported in the literature, where a silane-based additive with radical vinyl groups was used [87]. With the addition of OV-POSS to the TPV system and the increase in the OV-POSS/DCP ratio, significant improvements in mechanical properties were obtained with the increase in crosslink density values. The improvement in crosslink density of EPDM/PP TPVs in the presence of OV-POSS was evaluated via the Flory–Rehner approach. As seen from Table 3, independent from EPDM/PP loading ratio, all samples exhibited higher crosslink density values with the addition of OV-POSS compared with EPDM/PP TPVs, including TAC as a coagent. This can be attributed to the forming of crosslink bridges between the constituents via vinyl groups of OV-POSS during the dynamic vulcanization process.

Figure 8 shows the changes in elongation at break values EPDM/PP-based TPVs. It was observed that the elongation at break values increased in the presence of peroxide for all the EPDM/PP and EPDM/PP/Coagent(X) TPV systems compared to that of the EPDM/PP blends. This increase in the elongation at break values could be due to the finer dispersion of EPDM in the PP matrix due to the improved interfacial interaction between the components mentioned above. The relative decrements observed in elongation at break values with increased coagent/DCP ratio are due to increased crosslink density and partially larger particle sizes of the dispersed phase. Higher elongation at break values was observed in EPDM/PP TPV systems, including OV-POSS as a coagent, with high PP concentration (30/70 EPDM/PP). This can be attributed to the increase in the number of chain ends per unit volume due to the chain scission reactions occurring in the PP phase.

When the hardness values of EPDM/PP/Coagent(X) TPVs are examined, a similar trend was observed with the tensile strength and 100% modulus values, especially when OV-POSS was used as the coagent. This was due to the improvement in crosslink density of the EPDM/PP system in the presence of OV-POSS (Figure 9).

The variation in the compression set values of TPVs with respect to coagent type and coagaent/DCP ratios are shown in Figure 10. The ability of a material to recover after being compressed and exhibit a lower compression set value is one of the fundamental features of a high-performance TPV. The improvement or deterioration of the compression set value is related to the crosslink density of the EPDM phase in the EPDM/PP system. In addition, the compression set value is significantly affected by the molecular structure of the continuous thermoplastic phase. In the current study, the compression set values deteriorated as the PP ratio increased in the TPVs. Improved compression set values were obtained for EPDM/PP/Coagent(X) TPV systems due to the enhanced crosslink efficiency. This is particularly pronounced in the presence of OV-POSS as a coagent. It was predicted that DCP molecules provide the formation of OV-POSS radicals involved in crosslinking. Therefore, chain scission reactions caused by DCP are suppressed. In addition, it was also suggested that some extent of the physical crosslinks could occur with the activation of OV-POSS. With the increasing OV-POSS concentration, the number of OV-POSS radicals increases; therefore, the amount of physical crosslinking also increases. This led to an improvement in compression set values. Moreover, the possible formation of an EPDM-OVPOSS-PP grafted structure on the EPDM and PP polymer chains leads to the formation of physical entanglements. Thus, it provides EPDM/PP system with lower compression set values.

### 3.3. Thermal Aging Resistance of the TPVs

In this section, the mechanical properties of the samples aged at 70 °C for 70 h were evaluated by compression tests. Table 4 shows the % changes in the compression set values of the blends and TPV systems with respect to coagent type and crosslinking system before and after aging. As seen from Table 4, the heat aging resistance of TPVs having OV-POSS as a coagent was higher than that of EPDM/PP blends both at 70 °C for 70 h and at room temperature for 22 h. The aging resistance of thermoplastic vulcanizates directly depends on the strong C-C bonds of TPVs. Moreover, with the increase in PP concentration in the structure of the EPDM/PP system, the aging resistance improves because the heat aging resistance of PP is higher than EPDM. On the other hand, with the increase in EPDM concentration, forming the network structure due to crosslinking with peroxide in TPVs also improves the heat aging resistance [62,88,89]. It can be postulated that the participation of OV-POSS in the network structure of EPDM/PP TPVs during dynamic vulcanization enhanced the thermal stability of EPDM/PP, which resulted in improved compression set values after aging.

### 3.4. Oil Resistance of the TPVs

**Figure 11:**
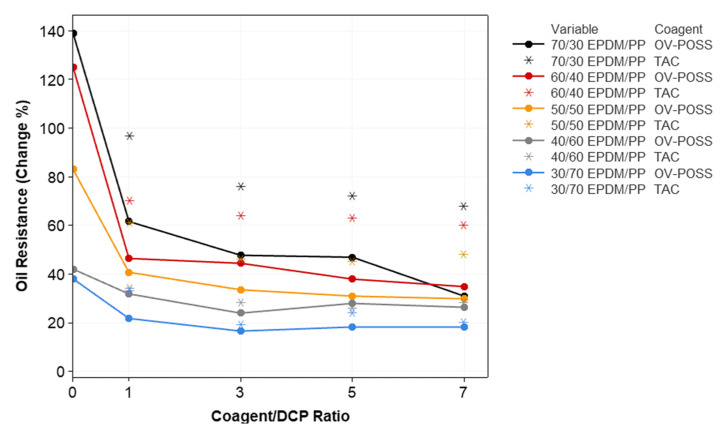
The variation in the oil resistance values of EPDM/PP TPVs with respect to EPDM/PP ratios, coagent types, and crosslinker system ratios.

The mass change of samples measured after the swelling test at 125 °C in IRM 903 standard oil for 70 h is given in Figure 11. Oil resistance in TPVs largely depends on the degree of the crosslinking of EPDM in EPDM/PP system and the formation of a three-dimensional network structure that resists oil penetration into the matrix. In addition, the increase in the crystallinity of the PP phase is one of the factors that improve oil resistance [62,90,91,92].

Figure 11 shows the variation of oil resistance values of the blends and TPV systems with respect to coagent types and crosslinking system ratios. As a comparison with EPDM/PP blends, the oil resistance of TPVs was improved regardless of the coagent type. Moreover, TPVs, including the highest content of PP, exhibited better oil resistance. This improvement was due to the increased crystallinity of PP in the presence of EPDM in the structure and the good oil resistance of PP. When the coagent types are compared, the oil resistance values of TPVs prepared with OV-POSS coagent are higher compared to TAC coagent. This was due to the increased crosslink density of TPVs and decreased particle sizes of the crosslinked dispersed phase of EPDM, which improved the interfacial interaction between the components in the presence of OV-POSS molecules.

### 3.5. Morphological Analysis by SEM

**Figure 12:**
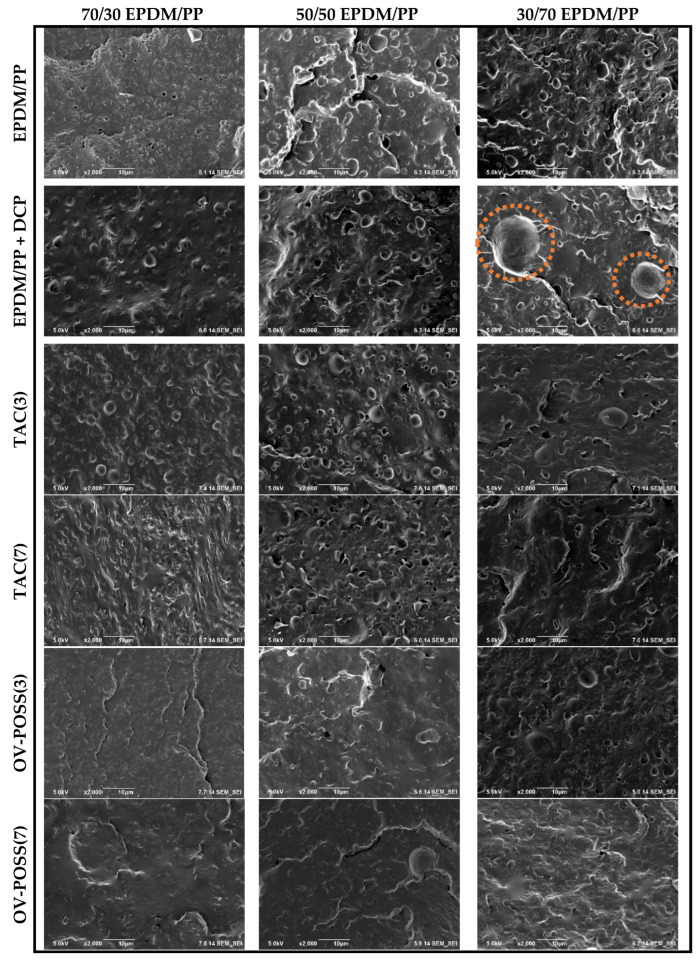
SEM pictures of 70/30, 50/50, and 30/70 EPDM/PP REF blends and TPVs (×2000, scale: 10 microns).

Figure 12 shows the SEM pictures obtained from cryogenically fractured surfaces of 70/30, 50/50, and 30/70 EPDM/PP REF blends and TPVs dynamically cured with DCP and containing a 0, 3, and 7 phr Coagent/DCP ratio. As shown in Figure 12, smooth surface morphology was observed regardless of the mixing ratio. For 70/30 EPDM/PP blend, spherical PP particles with an average particle size of 0.65 μm dispersed homogeneously in the matrix. However, when the EPDM concentration was increased to 50 phr, the average particle size of PP in both spherical and nodular structures increased. In 30/70 EPDM/PP blend, the average particle size of the dispersed phase of EPDM was found to be higher than that of 70/30 EPDM/PP system having a dispersed phase of PP. This was due to the higher viscosity of EPDM than that of PP. The presence of very large particles in the 30/70 EPDM/PP system after peroxide curing is remarkable. This is due to the fact that the addition of DCP to the 30/70 EPDM/PP blend resulted in chain scission of the PP phase; therefore, viscosity decreased and resulted in lower shear stress, which was necessary for the particle break-up process. Consequently, the coalescence of particles became more dominant than the break-up. Although similar findings were observed in vulcanizates with a high EPDM ratio, it was observed that the average particle size of the dispersed phase decreased. This can be attributed to the higher viscosity of EPDM and the consumption of peroxide for EPDM crosslinking rather than the chain scission of PP.

The effectiveness of the coagents used to increase the crosslinking efficiency can also be seen from the SEM pictures. As 7 phr TAC was added to the 70/30 EPDM/PP + DCP system, the average size of the dispersed EPDM particles decreased significantly and were homogeneously dispersed in the PP matrix. Additionally, the most notable result here is that the large spherical particles (circled in the SEM images) observed with the addition of DCP to the 30/70 EPDM/PP system are no longer visible. This indicates that the chain scission reactions occurring in the PP phase in the presence of DCP radicals are suppressed by the addition of TAC into the structure. For EPDM/PP TPVs having 7 phr OV-POSS coagent, almost a single-phase morphological structure was determined regardless of the mixing ratio. These findings show that the dispersed phase particle sizes decreased significantly depending on the composition of TPV in the presence of OV-POSS, indicating that OV-POSS is an effective coagent for PP/EPDM system.

### 3.6. Morphological Analysis by AFM

**Figure 13:**
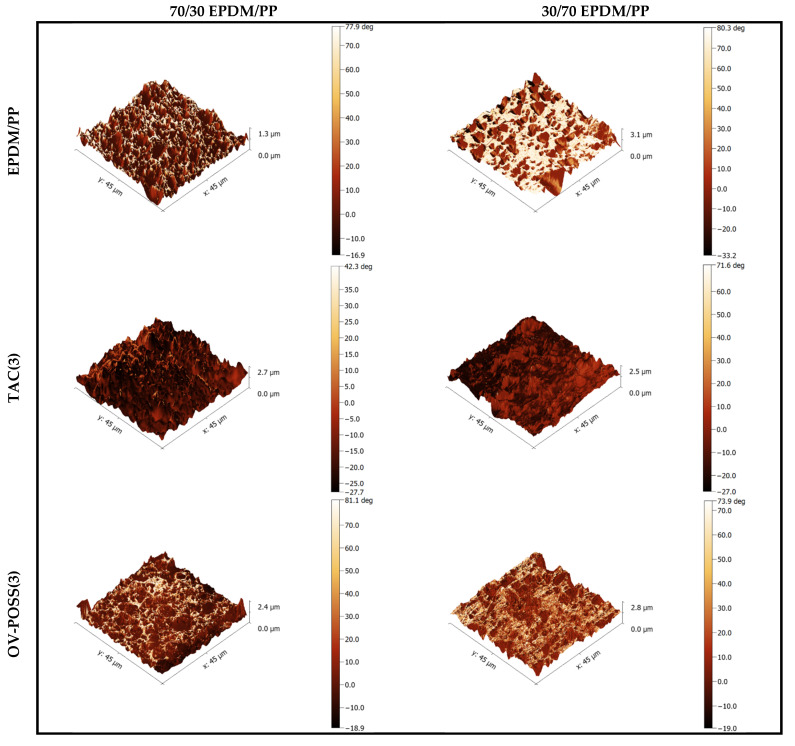
Three-dimensional AFM phase and topography images of 70/30 and 30/70 EPDM/PP and EPDM/PP/Coagent(3) TPVs.

**Figure 14:**
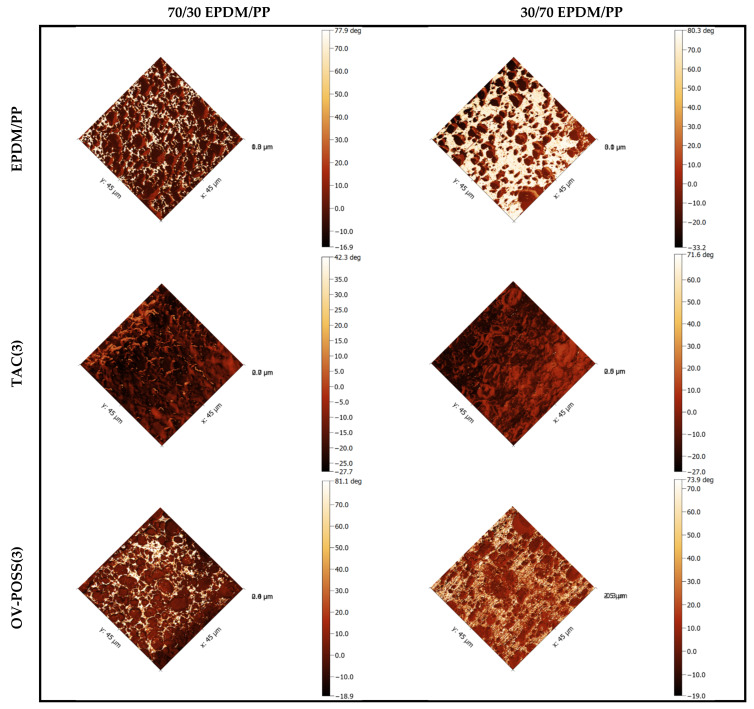
Two-dimensional AFM phase images of 70/30 and 30/70 EPDM/PP and EPDM/PP/Coagent(3) TPVs.

Two- and three-dimensional phase-topography AFM images of the samples are shown in Figure 13 and Figure 14. For EPDM/PP blends with high EPDM content, PP (white) was dispersed as small particles in the EPDM (brown) main phase before dynamic curing. For 30/70 EPDM/PP blends, the dispersed particle size of EPDM was much larger This was due to the fact that the viscosity of EPDM is higher than that of PP, as mentioned before.

When the AFM images of dynamically cured TPVs were examined, phase inversion in 70/30 EPDM/PP after vulcanization was observed. After phase inversion, the continuous EPDM phase turned into the dispersed phase and dispersed more homogeneously in the PP phase, resulting from EPDM’s crosslinking during dynamic vulcanization. It is clearly seen that the phase separation observed before dynamic curing is not present in EPDM/PP/Coagent(3) systems. This was because the chain scission reactions occurring in PP were partially prevented in the presence of a coagent, and hence, the cross-linked EPDM particles were finely dispersed. The smallest dispersed particle sizes in EPDM/PP system were obtained in the presence of OV-POSS in AFM pictures, which is another indication of the effectiveness of OV-POSS as a coagent for EPDM/PP vulcanizates.

## 4. Conclusions

In this study, the potential use of POSS nanocages having vinyl bonds was investigated as a coagent in EPDM/PP-based TPV production via dynamic vulcanization for the first time in the literature. The properties of the TPVs having POSS were compared with the TPVs produced using conventional coagents of TAC. The motivation for using POSS to prepare TPV was the potential of having increased crosslink density due to high functionality per POSS molecule and the limited segregation of the EPDM particles during dynamic vulcanization in the presence of POSS. In addition, once the OV-POSS forms the crosslink bridges between the constituents, then a self-reinforcing effect at a molecular level could be realized due to the stiff cage structure of POSS.

Rheological analyses showed that EPDM/PP TPVs, including OV-POSS as a coagent, exhibited higher complex viscosity values. The relaxation was suppressed, and the elasticity of the TPV became more dominant in the presence of OV-POSS. Mechanical test results revealed that the tensile strength and 100% modulus of EPDM/PP TPVs, including OV-POSS, was higher than that of EPDM/PP TPVs with TAC due to the higher crosslinking density in the presence of OV-POSS. SEM and AFM analysis showed that dispersed phase particle sizes decreased significantly depending on the mixing ratio and coagent type. In general, OV-POSS nanoparticles with unsaturated double bonds were found to be effective coagents for EPDM/PP systems compared to conventional coagents.

## Figures and Tables

**Table 1:** Materials used in the study.

Materials	Commercial Name and Manufacturer	Chemical Structure	Physical Properties and Descriptions
Polypropylene (PP)	Moplen HP456J, Lyondell Basel, Belgium	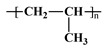	Homopolymer MFI: 3.4 (230 °C, 2.16 kg/10 min)
Ethylene Propylene Diene Monomer (EPDM)	Dutral TER 4548, Versalis, Germany	^ 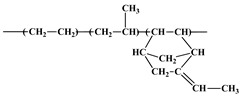 ^	Mooney Viscosity: 47 MU (1 + 4) 125 °C Ash content: max 0.3% Polypropylene content: 36% ENB content: 4.5% Oil content: 50% paraffin oil
Triallyl isocyanate (TAC)	TAC/GR 50, Kettlitz-Chemie GmbH & Co. KG. Germany	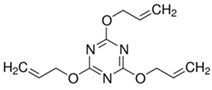	Density: 1.17 g/cm^3^ Ash content: 25.5% ± 1.5 Appearance: White, soft grain
Dicumyl peroxide (DCP)	Luperox DC40P, Arkema, France	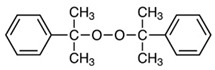	Molecular weight: 270.4 g/mol Peroxide content: 38–42% Active oxygen content: %2.25–2.49 Density: 0.38 g/cm^3^ Appearance: White powder
POSS	Octavinyl-POSS, Hybrid Plastics Inc., Hattiesburg, MS, USA	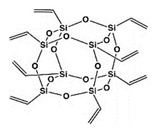	Molecular weight: 632.31 g/mol Soluble in: THF, Chloroform Appearance: White powder

**Table 2:** Abbreviation of samples.

EPDM * (phr)	PP (phr)	DCP (phr)	X (Coagent/DCP Ratio)	Antioxidants (phr)	Abbreviation
140	30	1.75	0	0.3	70/30 EPDM/PP/Coagent(X)
120	40	1.50	1	0.4	60/40 EPDM/PP/Coagent(X)
100	50	1.25	3	0.5	50/50 EPDM/PP/Coagent(X)
80	60	1.00	5	0.6	40/60 EPDM/PP/Coagent(X)
60	70	0.75	7	0.7	30/70 EPDM/PP/Coagent(X)

* EPDM includes 50% paraffin oil.

**Table 3:** Flory–Rehner results of TPVs with respect to the coagent types and crosslinker system ratios.

		70/30	60/40	50/50	40/60	30/70
**Crosslink density (mol/m^3^)**	**EPDM/PP + DCP**	103.0 ± 3.5	130.8 ± 9.2	208.7 ± 15.0	324.8 ± 29.1	462.4 ± 24.8
	**EPDM/PP/TAC(1)**	230.4 ± 18.9	250.0 ± 12.8	316.4 ± 21.1	393.5 ± 21.0	479.6 ± 19.0
	**EPDM/PP/TAC(3)**	238.7 ± 17.2	264.6 ± 14.6	334.3 ± 13.9	422.8 ± 16.6	505.2 ± 20.5
	**EPDM/PP/TAC(5)**	239.8 ± 15.6	283.3 ± 18.5	354.5 ± 15.2	437.0 ± 12.2	582.1 ± 27.0
	**EPDM/PP/TAC(7)**	245.1 ± 10.5	323.9 ± 24.2	380.9 ± 11.5	467.5 ± 18.6	601.4 ± 13.2
	**EPDM/PP/OV-POSS(1)**	280.1 ± 16.6	319.3 ± 24.1	393.7 ± 19.5	477.8 ± 13.5	608.8 ± 36.2
	**EPDM/PP/OV-POSS(3)**	328.2 ± 25.6	386.5 ± 17.8	422.8 ± 20.5	559.3 ± 35.4	748.9 ± 40.8
	**EPDM/PP/OV-POSS(5)**	411.4 ± 14.5	484.9 ± 18.2	551.7 ± 19.0	658.6 ± 18.0	778.8 ± 41.3
	**EPDM/PP/OV-POSS(7)**	511.6 ± 13.2	558.0 ± 34.7	661.6 ± 25.0	798.4 ± 34.8	806.6 ± 50.6

**Table 4:** The % changes in the compression set values of the blends and TPV systems with respect to coagent types and crosslinker system ratios after aging.

		70/30	60/40	50/50	40/60	30/70
**Compression Set (%)** **(70 h. 70 °C)**	**EPDM/PP**	81.1 ± 2.0	82.9 ± 1.1	84.0 ± 0.3	85.8 ± 7.1	87.0 ± 4.2
	**EPDM/PP + DCP**	52.7 ± 1.8	56.5 ± 2.1	57.5 ± 1.3	62.9 ± 1.1	71.7 ± 1.5
	**EPDM/PP/TAC(1)**	50.5 ± 0.1	57.7 ± 0.5	62.8 ± 0.3	65.5 ± 4.1	73.0 ± 1.1
	**EPDM/PP/TAC(3)**	48.1 ± 0.8	53.3 ± 2.1	62.2 ± 0.9	65.3 ± 0.3	72.7 ± 1.9
	**EPDM/PP/TAC(5)**	47.3 ± 1.2	52.4 ± 0.8	58.5 ± 0.9	65.2 ± 0.8	71.6 ± 0.6
	**EPDM/PP/TAC(7)**	45.8 ± 1.7	47.9 ± 1.5	57.9 ± 1.4	64.9 ± 0.4	69.2 ± 2.6
	**EPDM/PP/OV-POSS(1)**	40.6 ± 3.9	46.4 ± 0.2	53.9 ± 1.1	61.0 ± 1.9	70.6 ± 3.0
	**EPDM/PP/OV-POSS(3)**	39.6 ± 1.3	45.5 ± 1.7	52.0 ± 0.6	60.4 ± 0.3	69.5 ± 0.1
	**EPDM/PP/OV-POSS(5)**	39.1 ± 0.7	44.1 ± 1.6	51.8 ± 1.4	59.8 ± 0.2	69.4 ± 1.4
	**EPDM/PP/OV-POSS(7)**	38.6 ± 3.6	43.5 ± 1.5	51.0 ± 1.5	59.5 ± 0.5	65.9 ± 2.0
**Changes (%)**	**EPDM/PP**	68	63	64	63	54
	**EPDM/PP + DCP**	58	37	15	18	25
	**EPDM/PP/TAC(1)**	52	46	34	32	29
	**EPDM/PP/TAC(3)**	58	44	45	37	30
	**EPDM/PP/TAC(5)**	57	43	44	45	36
	**EPDM/PP/TAC(7)**	68	50	55	45	32
	**EPDM/PP/OV-POSS(1)**	35	26	33	36	34
	**EPDM/PP/OV-POSS(3)**	46	42	39	35	33
	**EPDM/PP/OV-POSS(5)**	34	28	38	44	49
	**EPDM/PP/OV-POSS(7)**	41	29	43	44	48

## Data Availability

The data presented in this study are available on request from the corresponding author.

## Supplementary Materials

The following supporting information can be downloaded at: https://www.mdpi.com/article/10.3390/polym15102267/s1, Figure S1: Complex viscosity of EPDM/PP TPVs as a function of EPDM/PP ratio and coagent types (a) EPDM/PP/TAC(3), (b) EPDM/PP/OV-POSS(3), (c) EPDM/PP/TAC(5) and (d) EPDM/PP/OV-POSS(5).; Figure S2: The storage modulus of EPDM/PP TPVs as a function of EPDM/PP ratio and coagent types (a) EPDM/PP/TAC(3), (b) EPDM/PP/OV-POSS(3), (c) EPDM/PP/TAC(5) and (d) EPDM/PP/OV-POSS(5).; Figure S3: The loss modulus of EPDM/PP TPVs as a function of EPDM/PP ratio and coagent types (a) EPDM/PP/TAC(3), (b) EPDM/PP/OV-POSS(3), (c) EPDM/PP/TAC(5) and (d) EPDM/PP/OV-POSS(5); Figure S4: Representative stress-strain curves of EPDM/PP and EPDM/PP TPVs as a function of EPDM/PP ratio and coagent types (a) EPDM/PP/TAC(1), (b) EPDM/PP/OV-POSS(1), (c) EPDM/PP/TAC(5), (d) EPDM/PP/OV-POSS(5), (e) EPDM/PP/TAC(7), and (f) EPDM/PP/OV-POSS(7).

## References

[1] Antunes C.F., van Duin M., Machado A.V. (2011). Morphology and Phase Inversion of EPDM/PP Blends—Effect of Viscosity and Elasticity. Polym. Test..

[2] Antunes C.F., Machado A.V., van Duin M. (2011). Morphology Development and Phase Inversion during Dynamic Vulcanisation of EPDM/PP Blends. Eur. Polym. J..

[3] Liang Y., Wang H., Li J., Wu S., Han W., Kang H., Fang Q. (2021). Green Thermoplastic Vulcanizates Based on Silicone Rubber and Poly(butylene succinate) via in situ Interfacial Compatibilization. ACS Omega.

[4] Nakason C., Wannavilai P., Kaesaman A. (2006). Effect of Vulcanization System on Properties of Thermoplastic Vulcanizates Based on Epoxidized Natural Rubber/Polypropylene Blends. Polym. Test..

[5] Nakason C., Jarnthong M., Kaesaman A., Kiatkamjornwong S. (2008). Thermoplastic Elastomers Based on Epoxidized Natural Rubber and High-Density Polyethylene Blends: Effect of Blend Compatibilizers on the Mechanical and Morphological Properties. J. Appl. Polym. Sci..

[6] Mani S., Cassagnau P., Bousmina M., Chaumont P. (2011). Morphology Development in Novel Composition of Thermoplastic Vulcanizates Based on PA12/PDMS Reactive Blends. Macromol. Mater. Eng..

[7] Tian M., Han J., Zou H., Tian H., Wu H., She Q., Chen W., Zhang L. (2012). Dramatic Influence of Compatibility on Crystallization Behavior and Morphology of Polypropylene in NBR/PP Thermoplastic Vulcanizates. J. Polym. Res..

[8] Yao P., Wu H., Ning N., Zhang L., Tian H., Wu Y., Hu G.-H., Chan T.W., Tian M. (2016). Microstructure and Properties of Bromo-Isobutylene–Isoprene Rubber/Polyamide 12 Thermoplastic Vulcanizate toward Recyclable Inner Liners for Green Tires. RSC Adv..

[9] Babu R.R., Naskar K. (2010). Recent Developments on Thermoplastic Elastomers by Dynamic Vulcanization. Advanced Rubber Composites.

[10] Nakason C., Nuansomsri K., Kaesaman A., Kiatkamjornwong S. (2006). Dynamic Vulcanization of Natural Rubber/High-Density Polyethylene Blends: Effect of Compatibilization, Blend Ratio and Curing System. Polym. Test..

[11] Nakason C., Jarnthong M., Kaesaman A., Kiatkamjornwong S. (2009). Influences of Blend Proportions and Curing Systems on Dynamic, Mechanical, and Morphological Properties of Dynamically Cured Epoxidized Natural Rubber/High-Density Polyethylene Blends. Polym. Eng. Sci..

[12] Nakason C., Jamjinno S., Kaesaman A., Kiatkamjornwong S. (2008). Thermoplastic Elastomer Based on High-Density Polyethylene/Natural Rubber Blends: Rheological, Thermal, and Morphological Properties. Polym. Adv. Technol..

[13] George J., Neelakantan N.R., Varughese K.T., Thomas S. (2006). Failure Properties of Thermoplastic Elastomers from Polyethylene/Nitrile Rubber Blends: Effect of Blend Ratio, Dynamic Vulcanization, and Filler Incorporation. J. Appl. Polym. Sci..

[14] Soares B.G., Almeida M.S.M., Deepa Urs M.V., Kumaraswamy G.N., Ranganathaiah C., Siddaramaiah, Mauler R. (2006). Influence of Curing Agent and Compatibilizer on the Physicomechanical Properties of Polypropylene/Nitrile Butadiene Rubber Blends Investigated by Positron Annihilation Lifetime Technique. J. Appl. Polym. Sci..

[15] Wu H., Ning N., Zhang L., Tian H., Wu Y., Tian M. (2013). Effect of Additives on the Morphology Evolution of EPDM/PP TPVs during Dynamic Vulcanization in a Twin-Screw Extruder. J. Polym. Res..

[16] Yao P., Wu H., Ning N., Zhang L., Tian H., Wu Y., Hu G., Chan T.W., Tian M. (2016). Properties and Unique Morphological Evolution of Dynamically Vulcanized Bromo-Isobutylene-Isoprene Rubber/Polypropylene Thermoplastic Elastomer. RSC Adv..

[17] Van Dyke J.D., Gnatowski M., Koutsandreas A., Burczyk A. (2003). A Study of Dynamic Vulcanization for Polyamide-12 and Chlorobutyl Rubber. J. Appl. Polym. Sci..

[18] Nicolini A., de Campos Rocha T.L.Á., Maldaner Jacobi M.A. (2008). Dynamically Vulcanized PP/EPDM Blends: Influence of Curing Agents on the Morphology Evolution. J. Appl. Polym. Sci..

[19] Patermann S., Altstädt V. (2015). Influence of Different Crosslinking Systems on the Mechanical and Morphological Properties of Thermoplastic Vulcanizates. AIP Conference Proceedings.

[20] Coran A.Y., Patel R. (1980). Rubber-Thermoplastic Compositions. Part I. EPDM-Polypropylene Thermoplastic Vulcanizates. Rubber Chem. Technol..

[21] Ezzati P., Ghasemi I., Azizi H., Karrabi M. (2008). Correclation between the Rheological Behaviour and Morphologies of PP/EPDM Blends in Various Dynamic Vulcanization Systems. Iran. Polym. J..

[22] Wang R., Peng Z., Fan P.P. (2011). Effect of Peroxide Content on Morphology and Properties of Thermoplastic Vulcanizates Based on PP and NR. Adv. Mater. Res..

[23] Varghese S., Alex R., Kuriakose B. (2004). Natural Rubber-Isotactic Polypropylene Thermoplastic Blends. J. Appl. Polym. Sci..

[24] Thitithammawong A., Nakason C., Sahakaro K., Noordermeer J. (2007). Effect of Different Types of Peroxides on Rheological, Mechanical, and Morphological Properties of Thermoplastic Vulcanizates Based on Natural Rubber/Polypropylene Blends. Polym. Test..

[25] Thitithammawong A., Nakason C., Sahakaro K., Noordermeer J.W.M. (2007). NR/PP Thermoplastic Vulcanizates: Selection of Optimal Peroxide Type and Concentration in Relation to Mixing Conditions. J. Appl. Polym. Sci..

[26] Endstra W.C. Aspects of Ethylene-Propylene Based Polymersi. Proceedings of the International Conference on ìVarious.

[27] Henning S.K., Boye W.M. (2009). Fundamentals of Curing Elastomers with Peroxides and Coagents II: Understanding the Relationship Between Coagent and Elastomer. Rubber World.

[28] Dikland H.G., Ruardy T., van der Does L., Bantjes A. (1993). New Coagents in Peroxide Vulcanization of EPM. Rubber Chem. Technol..

[29] Cao L., Jiang X., Ding J., Chen Y. (2015). Effect of Zinc Dimethacrylate on Compatibilization and Reinforcement of Peroxide Dynamically Cured PP/EPDM TPVs. J. Bulg. Chem. Commun..

[30] de Risi F.R., Noordermeer J.W.M. (2007). Effect of Methacrylate Co-Agents on Peroxide Cured PP/EPDM Thermoplastic Vulcanizates. Rubber Chem. Technol..

[31] Kodal M. (2018). Dinamik Olarak Vulkanize Edilmiş PP/SR Harmanlarının Mekanik, Isıl ve Morfolojik Özelliklerinin Incelenmesi. DÜMF Mühendislik Derg..

[32] Mali M., Kadam P., Mhaske S. (2017). Preparation and Characterization of Vinyltrimethoxysilane and Dicumyl Peroxide–Cured (Ethylene Propylene Diene Monomer)/Polypropylene Thermoplastic Vulcanizates. J. Vinyl Addit. Technol..

[33] Naskar K., Noordermeer J. (2003). Dynamically Vulcanized PP/EPDM Blends: Effects of Different Types of Peroxides on the Properties. Rubber Chem. Technol..

[34] Turan D., Sirin H., Ozkoc G. (2011). Effects of POSS Particles on the Mechanical, Thermal, and Morphological Properties of PLA and Plasticised PLA. J. Appl. Polym. Sci..

[35] Kodal M., Sirin H., Ozkoc G. (2014). Effects of Reactive and Nonreactive POSS Types on the Mechanical, Thermal, and Morphological Properties of Plasticized Poly(Lactic Acid). Polym. Eng. Sci..

[36] Kodal M. (2016). Polypropylene/Polyamide 6/POSS Ternary Nanocomposites: Effects of POSS Nanoparticles on the Compatibility. Polymer.

[37] Sirin H., Turan D., Ozkoc G., Gurdag S. (2013). POSS Reinforced PET Based Composite Fibers: “Effect of POSS Type and Loading Level”. Compos. Part B Eng..

[38] Sirin H., Kodal M., Karaagac B., Ozkoc G. (2016). Effects of Octamaleamic Acid-POSS Used as the Adhesion Enhancer on the Properties of Silicone Rubber/Silica Nanocomposites. Compos. Part B Eng..

[39] Kodal M., Şirin H., Karaağaç B., Özkoç G. (2020). Improved Interfacial Adhesion with the Help of Functional Polyhedral Oligomeric Silsesquioxanes in Silicone Rubber/Rayon Fiber Composites: Physical, Mechanical, Thermal, and Morphological Properties. Polym. Eng. Sci..

[40] Kilic Tuccar N., Can B.N., Kodal M., Ozkoc G. (2020). The Potential Use of Epoxy-POSS as a Reactive Hybrid Compatibilizers for PLA/PBAT Blends: “Effect of PBAT Molecular Weight and POSS Type”. Polym. Eng. Sci..

[41] Yazıcı N., Dursun S., Yarıcı T., Kılıç B., Mert O., Karaağaç B., Özkoç G., Kodal M. (2020). Effect of Octavinyl-Polyhedral Oligomeric Silsesquioxane on the Cross-Linking, Cure Kinetics, and Adhesion Properties of Natural Rubber/Textile Cord Composites. Ind. Eng. Chem. Res..

[42] Yazıcı N., Dursun S., Yarıcı T., Kılıç B., Arıcan M.O., Mert O., Karaağaç B., Özkoç G., Kodal M. (2021). The Outstanding Interfacial Adhesion between Acrylo-POSS/Natural Rubber Composites and Polyamide-Based Cords: ‘An Environmentally Friendly Alternative to Resorcinol-Formaldehyde Latex Coating’. Polymer.

[43] Turgut G., Dogan M., Tayfun U., Ozkoc G. (2018). The Effects of POSS Particles on the Flame Retardancy of Intumescent Polypropylene Composites and the Structure-Property Relationship. Polym. Degrad. Stab..

[44] Ozimek J., Pielichowski K. (2022). Preparation, Microstructure, and Microstructure-Properties Recent Advances in Polyurethane/POSS Hybrids for Biomedical Applications. Molecules.

[45] Ayendele E., Sarkar B., Alexandridis P. (2012). Polyhedral Oligomeric Silsesquioxane (POSS)-Containing Nanocomposites. Nanomaterials.

[46] Wang M., Chi H., Joshy K.S., Wang F. (2019). Progress in the Synthesis of Bifunctionalized Polyhedral Oligomeric Silsesquioxane. Polymers.

[47] Blanco I., Zaharescu T. (2022). The Effect of Polyhedral Oligomeric Silsesquioxanes (POSSs) Incorporation in Ethylene-propylene-diene-terpolymer (EPDM): A Thermal Study. J. Therm. Anal. Calorim..

[48] Ma X., Ji T., Zhang J., Shen S., Wang S., Wang J., Hou X., Yang S., Ma X. (2023). A Double-decker Silsesquioxane of Norbornene and Performance of Crosslinking Reactive Modified EPDM Ablation Resistance Composites. Compos. Part A Appl. Sci. Manuf..

[49] Morici E., Di Bartolo A., Arrigo R., Dintcheva N.T. (2016). Double Bond-Functionalized POSS: Dispersion and Crosslinking in Polyethylene-Based Hybrid Obtained by Reactive Processing. Polym. Bull..

[50] Wu J., Wu Z.L., Yang H., Zheng Q. (2014). Crosslinking of Low Density Polyethylene with Octavinyl Polyhedral Oligomeric Silsesquioxane as the Crosslinker. RSC Adv..

[51] Bicer E., Demir G.K., Kodal M., Ozkoc G. (2020). Investigation of Shape Memory Behavior and Physical Properties of Crosslinked Low Density Polyethylene/OvPOSS/TAIC Composites. AIP Conference Proceedings.

[52] Demir G.K., Bicer E., Kodal M., Ozkoc G. (2020). Cross-Linked LLDPE Composites in the Presence of POSS Nanoparticles and Poly(Ethylene Glycol) Dimethacrylate Coagent: “Comparison of Physical Properties and Shape Memory Behaviour”. AIP Conference Proceedings.

[53] Vennemann N., Bökamp K., Bröker D. (2006). Crosslink Density of Peroxide Cured TPV. Macromol. Symp..

[54] Shafieizadegan Esfahani A.R., Abdollahi M.M., Katbab A.A. (2016). Effects of Compounding Procedure on Morphology Development, Melt Rheology, and Mechanical Properties of Nanoclay Reinforced Dynamically Vulcanized EPDM/Polypropylene Thermoplastic Vulcanizates. Polym. Eng. Sci..

[55] Flory P.J., Rehner J. (1943). Statistical Mechanics of Cross-linked Polymer Networks I. Rubberlike Elasticity. J. Chem. Phys..

[56] Flory P.J. (1953). Principles of Polymer Chemistry.

[57] Parker W.O., Ferrando A., Ferri D., Canepari V. (2007). Cross-Link Density of a Dispersed Rubber Measured by 129Xe Chemical Shift. Macromolecules.

[58] Goharpey F., Nazockdast H., Katbab A.A. (2005). Relationship between the Rheology and Morphology of Dynamically Vulcanized Thermoplastic Elastomers Based on EPDM/PP. Polym. Eng. Sci..

[59] Ferry J.D. (1980). Viscoelastic Properties of Polymers.

[60] D’Orazio L., Mancarella C., Martuscelli E., Polato F. (1991). Polypropylene/Ethylene-Co-Propylene Blends: Influence of Molecular Structure and Composition of EPR on Melt Rheology, Morphology and Impact Properties of Injection-Moulded Samples. Polymer.

[61] Mighri F., Huneault M.A., Ajji A., Ko G.H., Watanabe F. (2001). Rheology of EPR/PP Blends. J. Appl. Polym. Sci..

[62] Ning N., Li S., Wu H., Tian H., Yao P., HU G.-H., Tian M., Zhang L. (2018). Preparation, Microstructure, and Microstructure-Properties Relationship of Thermoplastic Vulcanizates (TPVs): A Review. Prog. Polym. Sci..

[63] Ma P., Xu P., Zhai Y., Dong W., Zhang Y., Chen M. (2015). Biobased Poly(Lactide)/Ethylene- Co -Vinyl Acetate Thermoplastic Vulcanizates: Morphology Evolution, Superior Properties, and Partial Degradability. ACS Sustain. Chem. Eng..

[64] Wu H., Tian M., Zhang L., Tian H., Wu Y., Ning N., Hu G.-H. (2016). Effect of Rubber Nanoparticle Agglomeration on Properties of Thermoplastic Vulcanizates during Dynamic Vulcanization. Polymers.

[65] Babu R.R., Singha N.K., Naskar K. (2010). Interrelationships of Morphology, Thermal and Mechanical Properties in Uncrosslinked and Dynamically Crosslinked PP/EOC and PP/EPDM Blends. Express Polym. Lett..

[66] Ning N., Hu L., Yao P., Wu H., Han J., Zhang L., Tian H., Tian M. (2016). Study on the Microstructure and Properties of Bromobutyl Rubber (BIIR)/Polyamide-12 (PA12) Thermoplastic Vulcanizates (TPVs). J. Appl. Polym. Sci..

[67] Mark J.E. (1982). Experimental Determinations of Crosslink Densities. Rubber Chem. Technol..

[68] Mark J.E., Erman B., Eirich F.R. (2005). Science and Technology of Rubber.

[69] Babu R.R., Singha N.K., Naskar K. (2009). Effects of Mixing Sequence on Peroxide Cured Polypropylene (PP)/Ethylene Octene Copolymer (EOC) Thermoplastic Vulcanizates (TPVs). Part. I. Morphological, Mechanical and Thermal Properties. J. Polym. Res..

[70] Babu R.R., Singha N.K., Naskar K. (2009). Dynamically Vulcanized Blends of Polypropylene and Ethylene-Octene Copolymer: Comparison of Different Peroxides on Mechanical, Thermal, and Morphological Characteristics. J. Appl. Polym. Sci..

[71] Fu S.-Y., Feng X.-Q., Lauke B., Mai Y.-W. (2008). Effects of Particle Size, Particle/Matrix Interface Adhesion and Particle Loading on Mechanical Properties of Particulate–Polymer Composites. Compos. Part B Eng..

[72] Zhu Z.-K., Yang Y., Yin J., Qi Z.-N. (1999). Preparation and Properties of Organosoluble Polyimide/Silica Hybrid Materials by Sol-Gel Process. J. Appl. Polym. Sci..

[73] Fu S.-Y., Lauke B. (1998). Characterization of Tensile Behaviour of Hybrid Short Glass Fibre/Calcite Particle/ABS Composites. Compos. Part A Appl. Sci. Manuf..

[74] Fu S.Y., Lauke B. (1997). Analysis of Mechanical Properties of Injection Molded Short Glass Fibre (SGF)/Calcite/ABS Composites. J. Mater. Sci. Technol..

[75] Amdouni N., Sautereau H., Gerard J.F. (1992). Epoxy Composites Based on Glass Beads: Mechanical Properties. J. Appl. Polym. Sci..

[76] Wang M., Berry C., Braden M., Bonfield W. (1998). Young’s and Shear Moduli of Ceramic Particle Filled Polyethylene. J. Mater. Sci. Mater. Med..

[77] Hsueh C.-H. (1989). Effects of Aspect Ratios of Ellipsoidal Inclusions on Elastic Stress Transfer of Ceramic Composites. J. Am. Ceram. Soc..

[78] Young R.J., Beaumont P.W.R. (1977). Effect of Composition Upon Fracture of Silica Particle-Filled Epoxy–Resin Composites. J. Mater. Sci..

[79] Pukanszky B., Voros G. (1993). Mechanism of Interfacial Interactions in Particulate Filled Composites. Compos. Interfaces.

[80] Nakamura Y., Yamaguchi M., Okubo M., Matsumoto T. (1992). Effects of Particle Size on Mechanical and Impact Properties of Epoxy Resin Filled with Spherical Silica. J. Appl. Polym. Sci..

[81] Reynaud E., Jouen T., Gauthier C., Vigier G., Varlet J. (2001). Nanofillers in Polymeric Matrix: A Study on Silica Reinforced PA6. Polymer.

[82] Ou Y., Yang F., Yu Z.-Z. (1998). A New Conception on the Toughness of Nylon 6/Silica Nanocomposite Prepared via in Situ Polymerization. J. Polym. Sci. Part B Polym. Phys..

[83] Liang J.Z., Li R.K.Y., Tjong S.C. (1997). Tensile Fracture Behaviour and Morphological Analysis of Glass Bead Filled Low Density Polyethylene Composite. Plast. Rubber Compos. Process. Appl..

[84] Varlet J., Cavaillé J.Y., Perez J., Johari G.P. (1990). Dynamic Mechanical Spectrometry of Nylon-12. J. Polym. Sci. Part B Polym. Phys..

[85] Tjong S.C., Xu S.A. (2001). Ternary Polymer Composites: PA6,6/Maleated SEBS/Glass Beads. J. Appl. Polym. Sci..

[86] Levita G., Marchetti A., Lazzeri A. (1989). Fracture of Ultrafine Calcium Carbonate/Polypropylene Composites. Polym. Compos..

[87] Mali M., Marathe A., Mhaske S. (2018). Influence of (Methacryloxymethyl)Methyldimethoxysilane on DCP Cured EPDM/PP Thermoplastic Vulcanizates. J. Vinyl Addit. Technol..

[88] Reffai Syed Ismail S.M., Chatterjee T., Naskar K. (2015). Development of Novel Polar Thermoplastic Vulcanizates Based on Ethylene Acrylic Elastomer and Polyamide 12 with Special Reference to Heat and Oil Aging. J. Appl. Polym. Sci..

[89] Saleesung T., Saeoui P., Sirisinha C. (2010). Mechanical and Thermal Properties of Thermoplastic Elastomer Based on Low Density Polyethylene and Ultra-Fine Fully-Vulcanized Acrylonitrile Butadiene Rubber Powder (UFNBRP). Polym. Test..

[90] Ning N., Hua Y., Wu H., Zhang L., Wu S., Tian M., Tian H., Hu G.-H. (2016). Novel Heat and Oil-Resistant Thermoplastic Vulcanizates Based on Ethylene-Vinyl Acetate Rubber/Poly(Vinylidene Fluoride). RSC Adv..

[91] Tian M., Han J., Wu H., Tian H., She Q., Chen W., Zhang L. (2012). Effect of the Compatibility on the Morphology and Properties of Acrylonitrile-Butadiene Rubber/Polypropylene Thermoplastic Vulcanizates. J. Appl. Polym. Sci..

[92] Dutta J., Ramachandran P., Naskar K. (2016). Scrutinizing the Influence of Peroxide Crosslinking of Dynamically Vulcanized EVA/TPU Blends with Special Reference to Cable Sheathing Applications. J. Appl. Polym. Sci..

