# Supplementary material for: Unlocking the Potential Use of Reactive POSS as a Coagent for EPDM/PP-Based TPV
Source: Polymers (Basel). 2023 May 11;15(10):2267. doi: 10.3390/polym15102267 (PMC10222900; doi:10.3390/polym15102267)
Supplement: Supplementary file 1 [file polymers-15-02267-s001.zip › polymers-2310978-supplementary.pdf]

Supplementary Material

Unlocking the Potential Use of Reactive POSS as a Coagent for EPDM/PP-Based TPV

Nazlı Yazıcı Çakır [1], Özgenur İnan [2], Merve Ergün [1], Mehmet Kodal [1,2,3,*] and Güralp Özkoç [3,4,5]

[1] Department of Chemical Engineering, Kocaeli University, 41001 Kocaeli, Turkey; nazliyazici93@gmail.com (N.Y.Ç.); mervemetin1576@gmail.com (M.E.)
[2] Polymer Science and Technology Graduate Program, Kocaeli University, 41001 Kocaeli, Turkey; inannozgee@gmail.com
[3] Nanotechnology Research and Application Center, Sabancı University, 34956 Istanbul, Turkey; guralp.ozkoc@istinye.edu.tr
[4] Department of Chemistry, Istinye University, 34396 Istanbul, Turkey
[5] Xplore Instruments B.V., 6135 KT Sittard, The Netherlands
* Correspondence: mehmet.kodal@kocaeli.edu.tr; Tel.: +90-262-303-3540

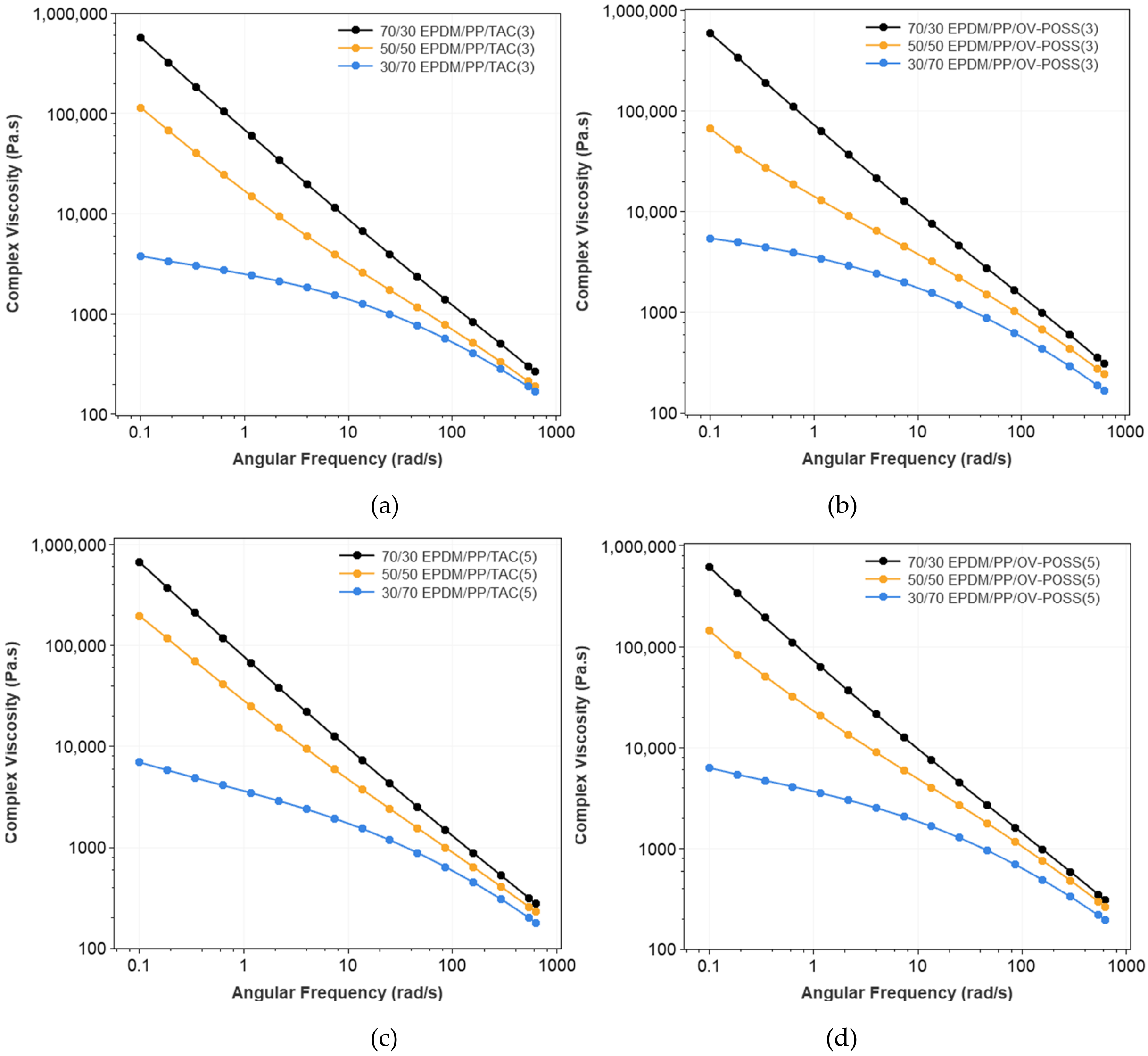

Figure S1. Complex viscosity of EPDM/PP TPVs as a function of EPDM/PP ratio and coagent types (a) EPDM/PP/TAC(3), (b) EPDM/PP/OV-POSS(3), (c) EPDM/PP/TAC(5) and (d) EPDM/PP/OV-POSS(5).

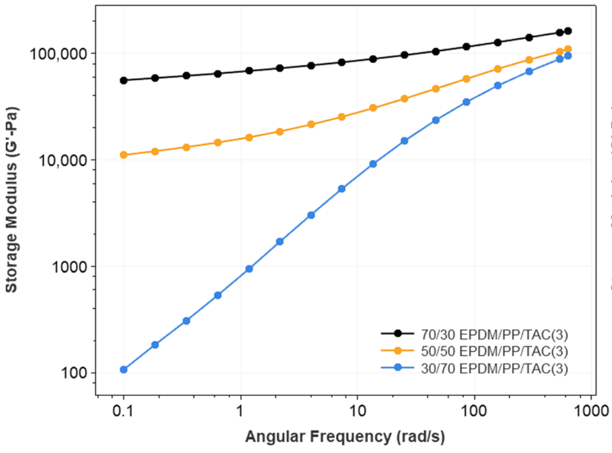

(a)

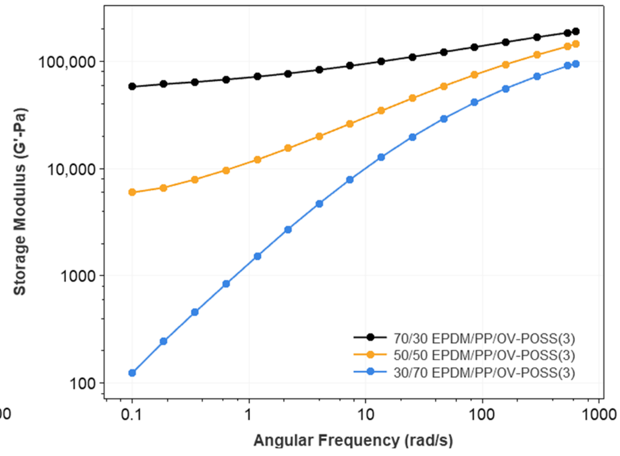

(b)

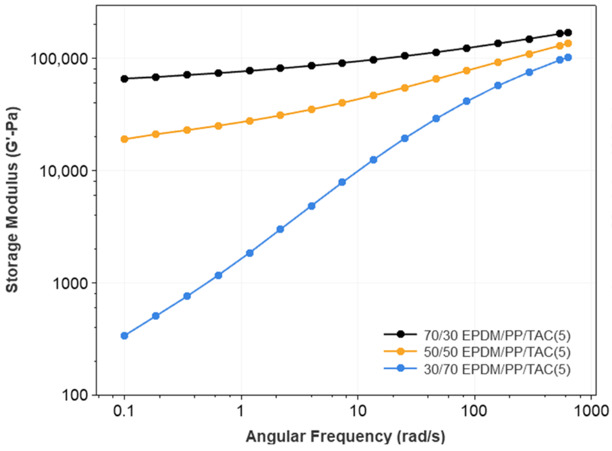

(c)

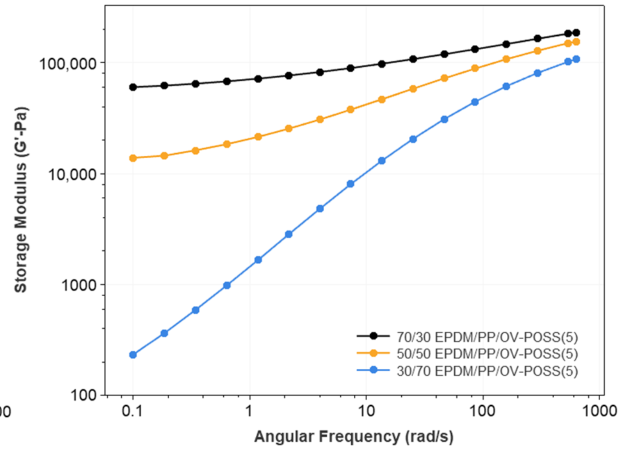

(d)

Figure S2. The storage modulus of EPDM/PP TPVs as a function of EPDM/PP ratio and coagent types (a) EPDM/PP/TAC(3), (b) EPDM/PP/OV-POSS(3), (c) EPDM/PP/TAC(5) and (d) EPDM/PP/OV-POSS(5).

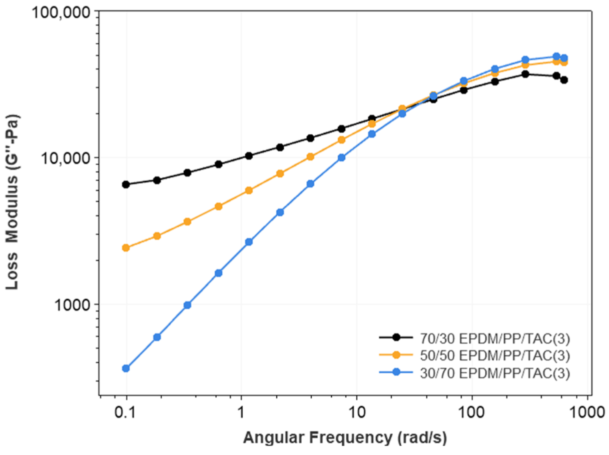

(a)

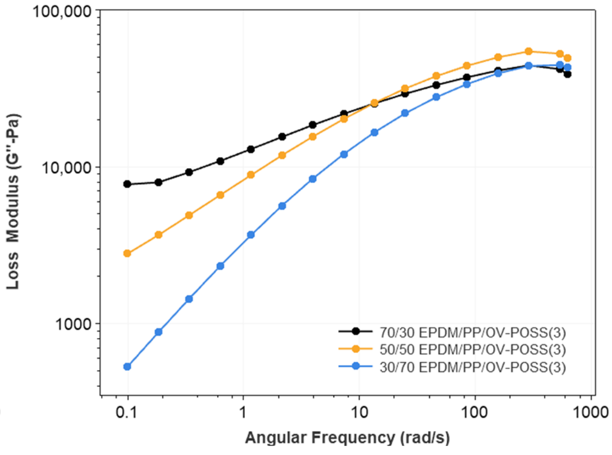

(b)

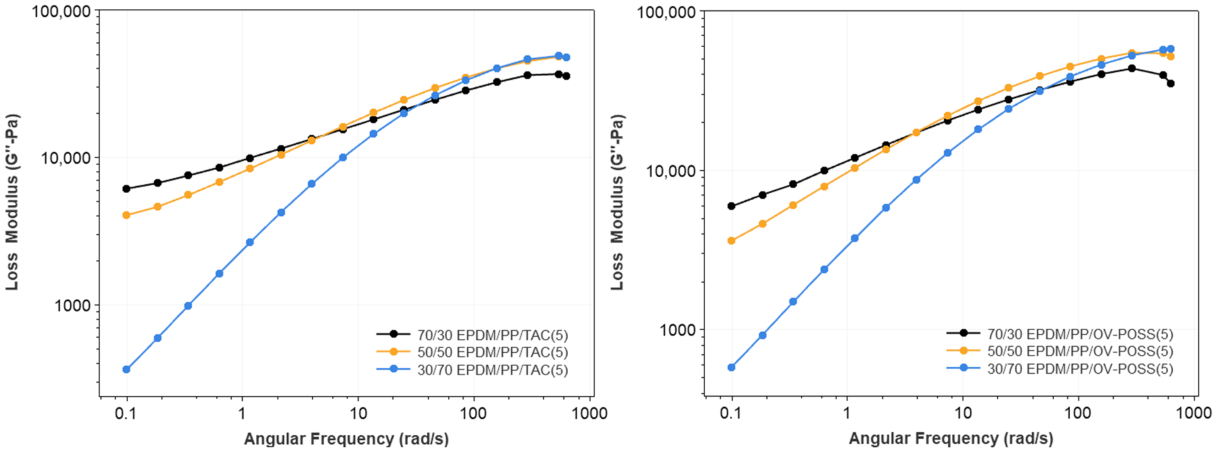

(c) (d)

Figure S3. The loss modulus of EPDM/PP TPVs as a function of EPDM/PP ratio and coagent types (a) EPDM/PP/TAC(3), (b) EPDM/PP/OV-POSS(3), (c) EPDM/PP/TAC(5) and (d) EPDM/PP/OV-POSS(5).

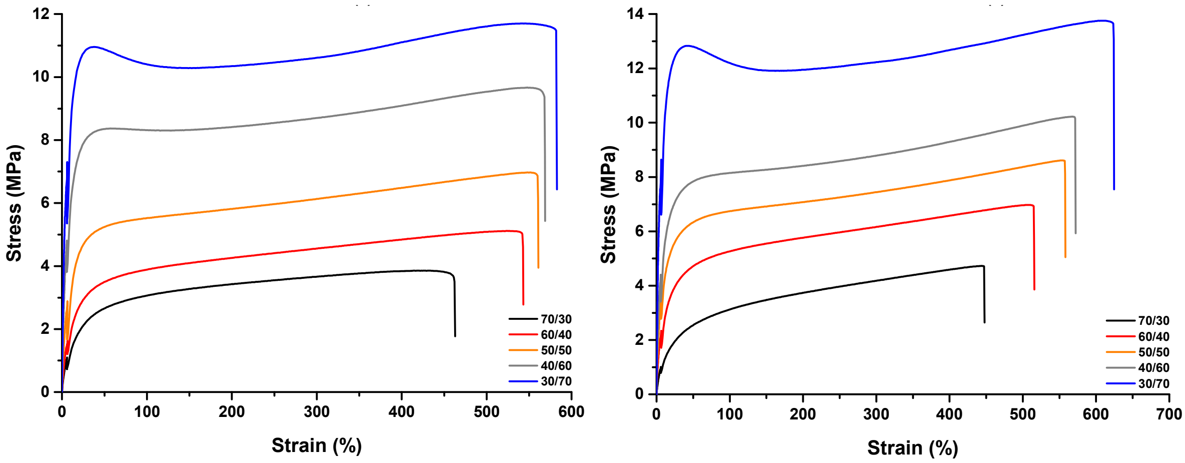

(a) (b)

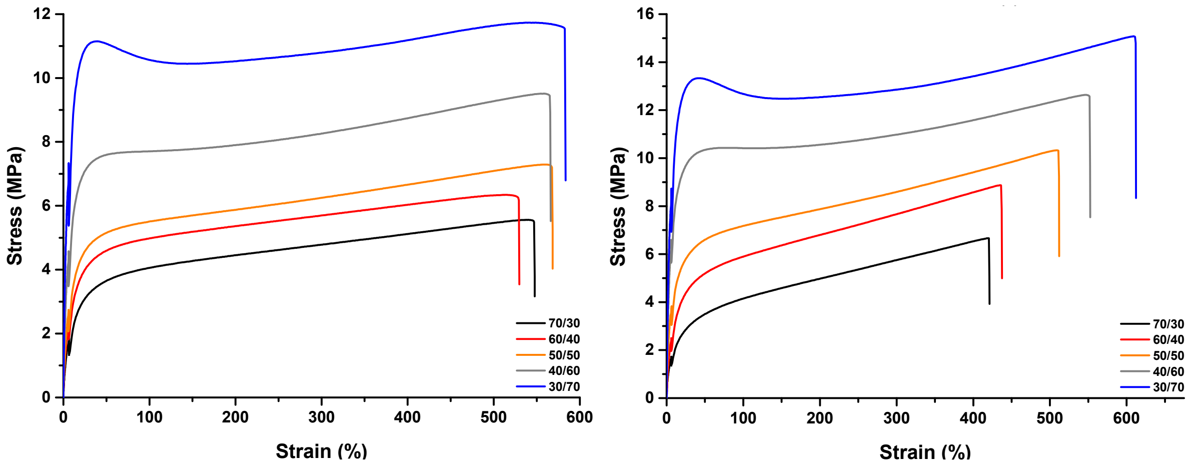

(c) (d)

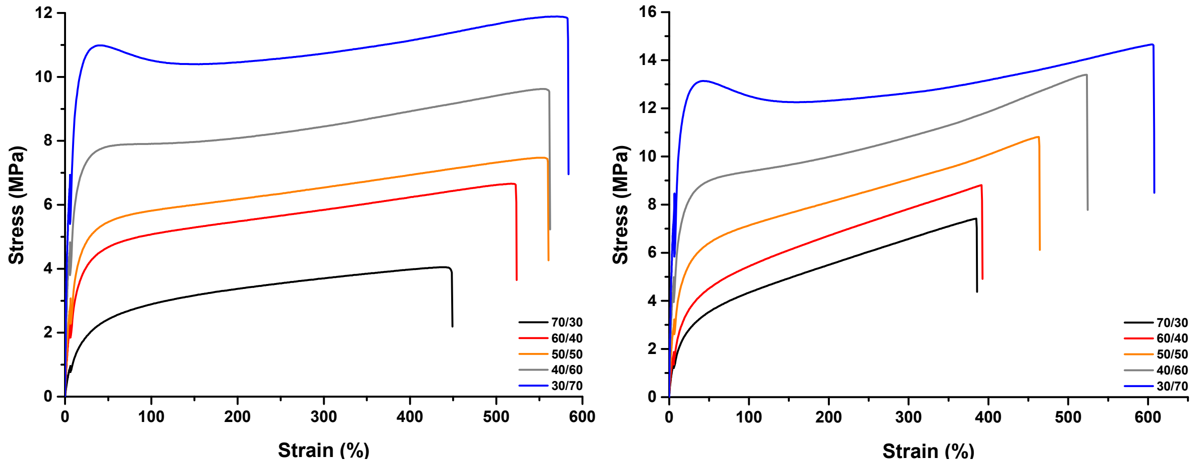

(e) (f)

Figure S4. Representative stress-strain curves of EPDM/PP and EPDM/PP TPVs as a function of EPDM/PP ratio and coagent types (a) EPDM/PP/TAC(1), (b) EPDM/PP/OV-POSS(1), (c) EPDM/PP/TAC(5), (d) EPDM/PP/OV-POSS(5), (e) EPDM/PP/TAC(7), and (f) EPDM/PP/OV-POSS(7).
